# *Yersinia pestis* Targets the Host Endosome Recycling Pathway during the Biogenesis of the *Yersinia*-Containing Vacuole To Avoid Killing by Macrophages

**DOI:** 10.1128/mBio.01800-17

**Published:** 2018-02-20

**Authors:** Michael G. Connor, Amanda R. Pulsifer, Donghoon Chung, Eric C. Rouchka, Brian K. Ceresa, Matthew B. Lawrenz

**Affiliations:** a Department of Microbiology & Immunology, University of Louisville School of Medicine, Louisville, Kentucky, USA; b Center for Predictive Medicine for Biodefense and Emerging Infectious Diseases, University of Louisville School of Medicine, Louisville, Kentucky, USA; c Department of Computer Engineering and Computer Science, University of Louisville, Louisville, Kentucky, USA; d Department of Pharmacology & Toxicology, University of Louisville School of Medicine, Louisville, Kentucky, USA; University of Michigan—Ann Arbor

**Keywords:** intracellular survival, plague, Rab GTPases, *Yersinia pestis*, endosome recycling

## Abstract

*Yersinia pestis* has evolved many strategies to evade the innate immune system. One of these strategies is the ability to survive within macrophages. Upon phagocytosis, *Y. pestis* prevents phagolysosome maturation and establishes a modified compartment termed the *Yersinia*-containing vacuole (YCV). *Y. pestis* actively inhibits the acidification of this compartment, and eventually, the YCV transitions from a tight-fitting vacuole into a spacious replicative vacuole. The mechanisms to generate the YCV have not been defined. However, we hypothesized that YCV biogenesis requires *Y. pestis* interactions with specific host factors to subvert normal vesicular trafficking. In order to identify these factors, we performed a genome-wide RNA interference (RNAi) screen to identify host factors required for *Y. pestis* survival in macrophages. This screen revealed that 71 host proteins are required for intracellular survival of *Y. pestis*. Of particular interest was the enrichment for genes involved in endosome recycling. Moreover, we demonstrated that *Y. pestis* actively recruits Rab4a and Rab11b to the YCV in a type three secretion system-independent manner, indicating remodeling of the YCV by *Y. pestis* to resemble a recycling endosome. While recruitment of Rab4a was necessary to inhibit YCV acidification and lysosomal fusion early during infection, Rab11b appeared to contribute to later stages of YCV biogenesis. We also discovered that *Y. pestis* disrupts global host endocytic recycling in macrophages, possibly through sequestration of Rab11b, and this process is required for bacterial replication. These data provide the first evidence that *Y. pestis* targets the host endocytic recycling pathway to avoid phagolysosomal maturation and generate the YCV.

## IMPORTANCE

*Yersinia pestis* can infect and survive within macrophages. However, the mechanisms that the bacterium use to subvert killing by these phagocytes have not been defined. To provide a better understanding of these mechanisms, we used an RNAi approach to identify host factors required for intracellular *Y. pestis* survival. This approach revealed that the host endocytic recycling pathway is essential for *Y. pestis* to avoid clearance by the macrophage. We further demonstrate that *Y. pestis* remodels the phagosome to resemble a recycling endosome, allowing the bacterium to avoid the normal phagolysosomal maturation pathway. Moreover, we show that infection with *Y. pestis* disrupts normal recycling in the macrophage and that disruption is required for bacterial replication. These findings provide the first evidence that *Y. pestis* targets the host endocytic recycling pathway in order to evade killing by macrophages.

## INTRODUCTION

*Yersinia pestis* is a facultative intracellular pathogen that causes the human disease known as plague (1, 2). There are three forms of human plague: bubonic, pneumonic, and septicemic. Each form of plague results in an acute infection that is delineated by the tissues primarily colonized by *Y. pestis*. Bubonic plague is the most common form and arises after a bite from a *Y. pestis*-infected flea. The bacteria rapidly disseminate from the inoculation site through the lymphatic system and colonize the draining lymph node (1, 3, 4). Eventually, the bacteria enter and replicate in the bloodstream, leading to septicemic plague (1, 5). In rare cases, *Y. pestis* can be directly inoculated into blood by a flea or from the bite of an infected animal, resulting in primary septicemic plague without colonization of the lymphatic system (1). From the blood, *Y. pestis* is distributed throughout the body and colonizes other tissues, such as the spleen, liver, and lungs. Colonization of the lungs leads to the development of secondary pneumonic plague and the potential for patients to aerosolize *Y. pestis* by coughing, and potential person-to-person transmission. Inhalation of infected aerosols by naive individuals can result in colonization of the lungs by *Y. pestis* and the development of primary pneumonic plague. All three forms of plague are very rapid infections with high mortality rates in the absence of early antibiotic treatment (1, 6). Furthermore, the ability for aerosol transmission of *Y. pestis* raises the potential for this bacterium to be used as a biological weapon (7).

*Y. pestis* is maintained through a zoonotic transmission cycle between rodents and fleas (1, 2). The ability of *Y. pestis* to exist in both the mammalian and flea hosts is a result of acquiring virulence factors required for the mammalian host and transmission factors required for flea colonization (1, 8–10). The bacterium regulates these factors to ensure expression of appropriate factors only when required (1, 8–12). Importantly, antiphagocytic factors expressed during mammalian infection, like the Ysc type three secretion system (T3SS), secreted Yop effectors, and the Caf1 capsule, are not required for flea infection, and thus are repressed in the flea vector (13, 14). Therefore, there is a transition period when *Y. pestis* is highly susceptible to recognition and phagocytosis by macrophages and neutrophils immediately upon flea transmission (15–17). This susceptibility was highlighted by intravital microscopy of the infection site by Shannon et al. (17). Following transmission of *Y. pestis* via the flea to the dermis of the ear, polymorphonuclear leukocytes (PMNs) are rapidly recruited to the infection site and appear to phagocytose the bacteria. To a lesser degree, host macrophages are also recruited and engulf bacteria, and infected macrophages appeared to migrate away from the infection site. Growing evidence suggests that these two phagocytes have very different abilities to kill *Y. pestis* (15, 17–22). Specifically, neutrophils appear to be more efficient at killing phagocytosed *Y. pestis* than macrophages (20–22). Moreover, several studies suggest that *Y. pestis* actively inhibits killing by both mouse and human macrophages (16, 19, 23–28).

The importance of intracellular *Y. pestis* survival in virulence has been highlighted by several studies. Ye et al. showed that animals depleted for macrophage/dendritic cell populations exhibited delayed *Y. pestis* dissemination and subsequently lower bacterial burdens during plague infection (29). Moreover, a *phoPQ* mutant, which is defective for survival within macrophages (23, 24, 30, 31), has a 75-fold attenuation in subcutaneous infection of BALB/c mice and a significant delay in the development of lethal disease in Swiss Webster mice (30, 31). While PhoPQ regulates many genes in *Y. pestis*, and thus may have pleiotropic effects during infection, many of the genes regulated by this operon have been shown to be important for stress response and intracellular survival, indicating that deficiency in intracellular survival of the *phoPQ* mutant contributes to the attenuated phenotypes (23). In contrast, *Y. pestis* is defective in intracellular survival in macrophages from canines and *Mus spretus* SEG mice, species that are relatively resistant to plague, compared to common laboratory murine macrophages, indicating that the ability of macrophages to kill *Y. pestis* may contribute to susceptibility to plague (32–34). These data, combined with studies showing the sensitivity of *Y. pestis* to PMN killing (20–22), suggest that *Y. pestis* infection of macrophages may provide an intracellular niche to avoid killing by PMNs during early stages of bubonic plague.

Upon phagocytosis by macrophages, *Y. pestis* actively inhibits phagosome-mediated killing (16, 23, 25–28). A hallmark of this process is the inhibition of phagosome acidification by *Y. pestis* (27). The bacterium remains within this phagosome throughout the course of the intracellular infection, eventually remodeling it into a compartment called the *Yersinia*-containing vacuole (YCV). In addition to maintaining a neutral pH, a subset of the YCVs eventually mature into autophagosome-like compartments, acquiring both LC3-II and double membranes (27). During late infection, the YCV expands from a tight-fitting vacuole to a spacious vacuole, coinciding with bacterial replication (23, 27, 33). Recently, we identified the first host factor required by *Y. pestis* for the YCV biogenesis process (35). The host GTPase Rab1b, which normally mediates endoplasmic reticulum (ER)-Golgi trafficking (36, 37), is rapidly recruited to the YCV and is required for *Y. pestis* to inhibit vacuole acidification and phagosome maturation. These data demonstrate that *Y. pestis* manipulates host factors to subvert phagosomal maturation and to generate a protective replicative niche within the macrophage. Here we describe a genome-wide, RNA interference (RNAi)-based high-throughput screen to identify additional host factors required for intracellular survival of *Y. pestis*. Network analysis of these genes revealed enrichment for host factors involved in the endocytic recycling pathway. We further show that *Y. pestis* actively recruits Rab4 and Rab11 to remodel the YCV to resemble host recycling endosomes in order to avoid phagolysosomal maturation in a T3SS-independent manner. Moreover, we demonstrate that *Y. pestis* infection also disrupts global host cell recycling, likely through sequestration of Rab11, and that disruption in recycling is important for intracellular replication.

## RESULTS

### *Y. pestis* requires host cell signal transduction, transport, and localization pathways to survive in macrophages.

**FIG 1:**
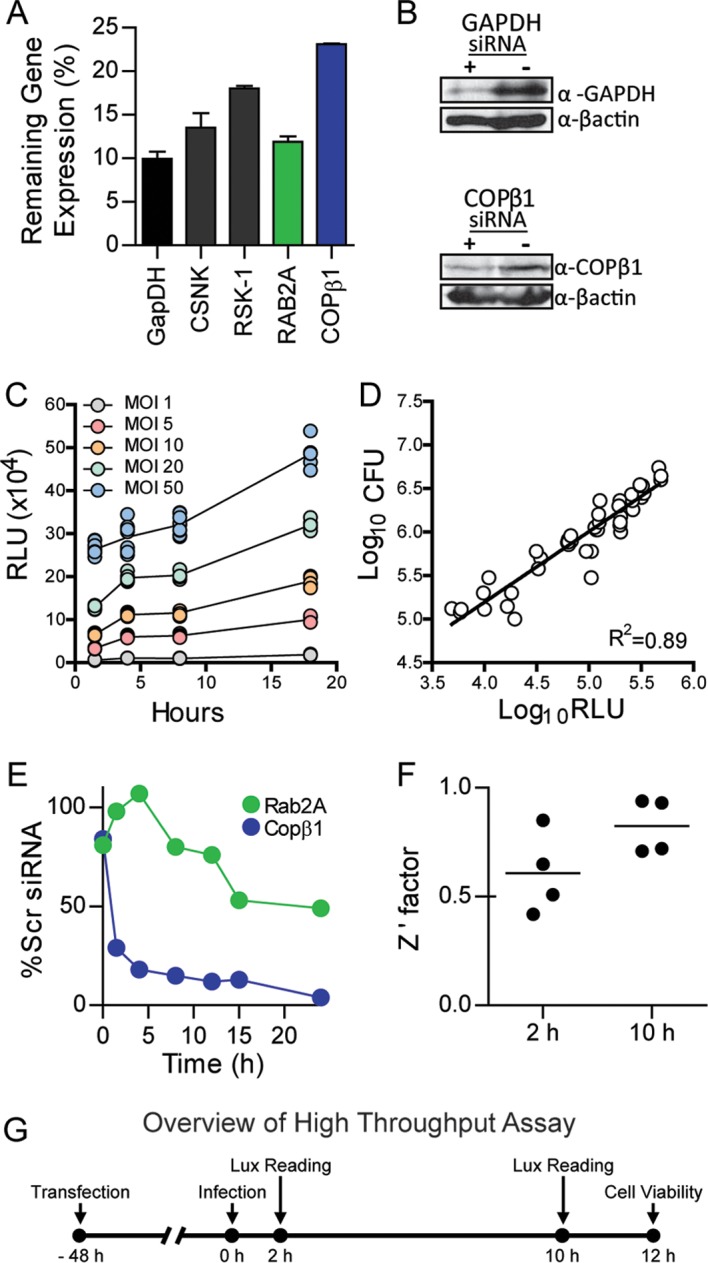
RNAi-based assay to identify host factors required for intracellular survival of *Y. pestis*. To determine whether reproducible RNAi could be achieved in RAW264.7 macrophages, cells were reverse transfected with siRNAs targeting indicated genes. (A and B) Forty-eight hours posttransfection, cells (*n* = 3) were harvested for RNA isolation and qRT-PCR (data represent the level of gene expression compared to the level for the scrambled siRNA control) (A) or protein isolation for Western blot analysis (β-actin was used as a loading control) (B). α-GAPDH, anti-GAPDH antibody; α-βactin, anti-βactin antibody. To demonstrate that the *Y. pestis* CO92 pCD1^(-)^ Lux_PtolC_ bioreporter accurately represents intracellular bacterial numbers, RAW264.7 macrophages were infected with *Y. pestis* CO92 pCD1^(-)^ Lux_PtolC_ at the indicated MOIs (*n* = 12), and extracellular bacteria were killed with gentamicin. (C) Bioluminescence (in relative light units [RLU]) of intracellular bacteria was determined at 1, 4, 8, and 18 h postinfection. (D) At 18 h, cells from each MOI (*n* = 3) were lysed, and bacterial numbers (CFU) were determined and compared to 18-h bioluminescence (in RLU). (E) To demonstrate that RNAi targeting specific genes could impact the intracellular survival of *Y. pestis*, RAW264.7 macrophages were transfected with siRNAs targeting Rab2A or COPβ1. Forty-eight hours posttransfection, macrophages were infected with *Y. pestis* CO92 pCD1^(-)^ Lux_PtolC_ (MOI of 10). Extracellular bacteria were killed with gentamicin, and intracellular bacterial bioluminescence was monitored over time. Data are represented as percent RLU of scrambled (Scr) siRNA control. (F) To demonstrate the robustness of the assay, RAW264.7 macrophages (*n* = 48) were reverse transfected with either scrambled siRNA (negative control) or siRNA targeting Copβ1 (positive control). Forty-eight hours posttransfection, macrophages were infected with *Y. pestis* CO92 pCD1^(-)^ Lux_PtolC_ (MOI of 10). Extracellular bacteria were killed with gentamicin, and intracellular bacterial bioluminescence was determined at 2 and 10 h postinfection. The Z’ factors from four independent experiments are shown (the bars are means). (G) Overview of optimized high-throughput assay for RNAi screening.

RNAi has been used to identify host factors required for intracellular survival of several pathogens (38–49). As macrophages are specifically infected during *Y. pestis* infection, our first goal was to select a macrophage cell line that was amendable to Lipofectamine-mediated transfection and RNAi necessary for high-throughput screening. Toward this end, we tested small interfering RNA (siRNA) transfection and knockdown in several human and mouse macrophage cell lines. While robust RNAi was observed in mouse macrophages, we were unable to reproducibly knock down gene expression in human cell lines (data not shown). On the basis of these results, we chose RAW264.7 mouse macrophages for further optimization. Using a combination of siRNAs targeting genes of variable expression levels, we optimized Lipofectamine/siRNA concentrations and transfection time to consistently achieve >70% knockdown of targets (Fig. 1A and B). Next, we infected cells transfected with Rab2 siRNA and Copβ1 siRNA (both siRNAs are known to inhibit intracellular survival of other pathogens [41, 42, 48]) with *Y. pestis* CO92 pCD1^(-)^ Lux_PtolC_, a bioluminescent bioreporter that can differentiate as little as a twofold difference in intracellular bacteria (Fig. 1C and D; *R*^2^ = 0.89) (50), to demonstrate that this bioreporter can be used to kinetically monitor changes in intracellular survival (Fig. 1E). Finally, using Copβ1 siRNA as a positive control, we calculated Z′ factor values for this assay of 0.61 and 0.83 at 2 and 10 h postinfection, respectively (Fig. 1F). Together, these data indicate a highly reproducible assay amenable to high-throughput screening (Fig. 1G).

**FIG 2:**
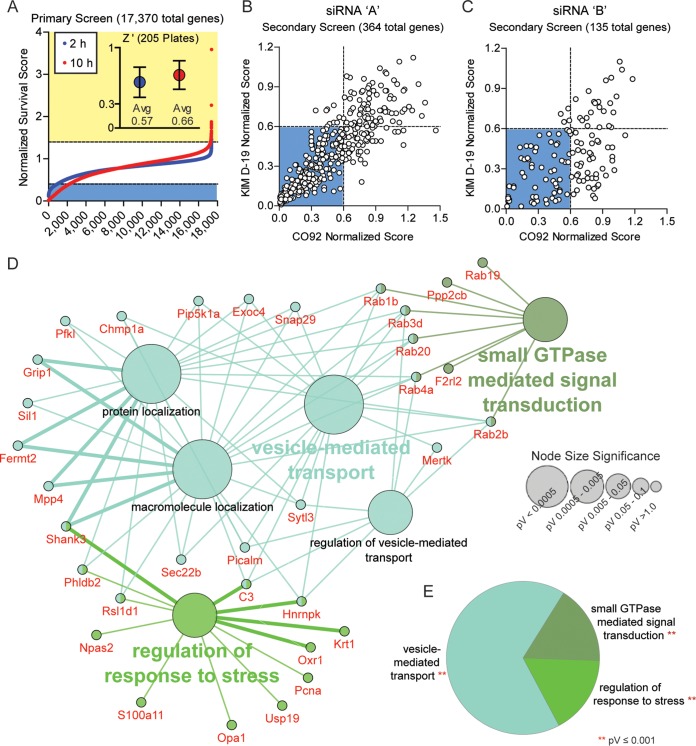
Identification of host factors required for intracellular survival of *Y. pestis*. RAW264.7 macrophages were reverse transfected with siRNAs for 48 h. (A) Transfected cells were infected with *Y. pestis* CO92 pCD1^(-)^ Lux_PtolC_ (MOI of 10), and intracellular bacterial bioluminescence (in RLU) was determined at 2 and 10 h postinfection. RLU values were normalized to the values for the controls and ranked from lowest to highest. Normalized scores of ≤0.4 are indicated by light blue shading, and normalized scores of ≥1.4 are indicated by yellow shading. (Inset) Average Z factor (Z′) ± standard deviation (SD) for all 205 screened plates. (B and C) For secondary validation, cells transfected with siRNA “A” (B) or siRNA “B” (C) were infected with *Y. pestis* CO92 pCD1^(-)^ Lux_PtolC_ or KIMD19 pCD1^(+)^ Lux_PtolC_ (MOI of 10), and intracellular bacterial bioluminescence (in RLU) was determined at 10 h postinfection. RLU values for each strain were normalized to the values for the controls and compared. Normalized scores of ≤0.6 are indicated by blue shading. (D) Cytoscape-generated layout for Gene Ontology (GO) term node clusters, with significant genes per cluster highlighted in red. Clusters are color coded by parent ontology, and subgroup ontology is labeled in black. Lines represent interconnections between detailed terms. Node size denotes significance. (E) Pie chart representing the percent parent ontology represented as a whole within validated genes. pV, *P* value.

Using this RNAi assay, 17,370 host genes were screened using a pooled siRNA approach (three siRNAs for each target were pooled into one well), and we monitored changes in intracellular survival of *Y. pestis*. Each plate also contained control wells transfected with scrambled or COPβ1 siRNAs. Bioluminescence was measured at 2 and 10 h postinfection, and Z′ factors were calculated from the control wells (the average Z′ factors for the screen at 2 and 10 h were 0.57 and 0.66, respectively). Bioluminescence was normalized for each plate based on control wells, and changes in intracellular survival of *Y. pestis* were ranked by normalized scores (Fig. 2A; see also Data Set S1 in the supplemental material). A total of 325 genes that inhibited bacterial growth and 39 genes that promoted bacterial growth were selected for secondary validation. For the secondary screen, a single siRNA (siRNA “A”) was used to validate the primary screen results. Furthermore, the primary hits were validated against two different *Y. pestis* strains, one with the Ysc type three secretion system (T3SS) (KIMD19 pCD1^(+)^ Lux_PtolC_) and one without it (CO92 pCD1^(-)^ Lux_PtolC_). A direct correlation was observed between the two strains (Fig. 2B; *r*_*s*_ = 0.87), supporting previous studies showing that the T3SS is dispensable for intracellular survival (16, 24, 35, 50). From the primary hits, 135 genes showed ≥40% inhibition of intracellular survival of *Y. pestis* and 7 showed a hypervirulent phenotype with ≥20% more growth than scrambled controls. These 142 genes were further screened using a second siRNA (siRNA “B”). Of the 142 genes, RNAi of 71 of these genes continued to show inhibition of intracellular *Y. pestis* survival, while one retained a hypervirulent phenotype (≥10% more growth than scrambled controls; Data Set S1 and Table S1).

10.1128/mBio.01800-17.7 TABLE S1  Host genes required for intracellular survival of *Yersinia pestis*. Host genes that passed secondary validation are listed by Entrez Gene identifier (ID), gene symbol, MGI gene or marker ID, and Ensembl ID. Download TABLE S1, PDF file, 0.1 MB. Copyright © 2018 Connor et al. 2018 Connor et al. This content is distributed under the terms of the Creative Commons Attribution 4.0 International license.

10.1128/mBio.01800-17.8 Data Set S1  Normalized data from primary and validation screens. Complete normalized survival data from the primary screen (“Primary Screen” tab) and the two validation screens (“Combined Validation Data” tab) are shown. Download Data Set S1, XLSX file, 1.2 MB. Copyright © 2018 Connor et al. 2018 Connor et al. This content is distributed under the terms of the Creative Commons Attribution 4.0 International license.

Gene Ontology (GO) clustering and network analyses of the 71 validated inhibition hits using all GO evidence codes, a minimum kappa score of 0.4, and a *P* value threshold of 0.05 revealed substantial clustering within the validated data set (Fig. 2D). Of the five enriched groups, the largest cluster was under vesicle-mediated transport (Fig. 2D and E; *P* ≤ 0.001). Under the parent GO term clusters, detailed GO terms significantly focused on host trafficking networks, with transport and localization as common themes. Additional enrichment included small-GTPase-mediated signal transduction and regulation of response to stress (Fig. 2D and E). Within the small GTPase signal trafficking, six Rab GTPases appeared to be required for intracellular survival of *Y. pestis*, and two trafficking pathways were highlighted: (i) endocytic recycling (Rab4a and Rab20) and (ii) retrograde trafficking (Rab1b and Rab2b) (51–56). Together, these data indicate targeting of specific host Rab-mediated signaling pathways by *Y. pestis* during infection of macrophages.

### Host cell recycling is essential for *Y. pestis* survival.

**FIG 3:**
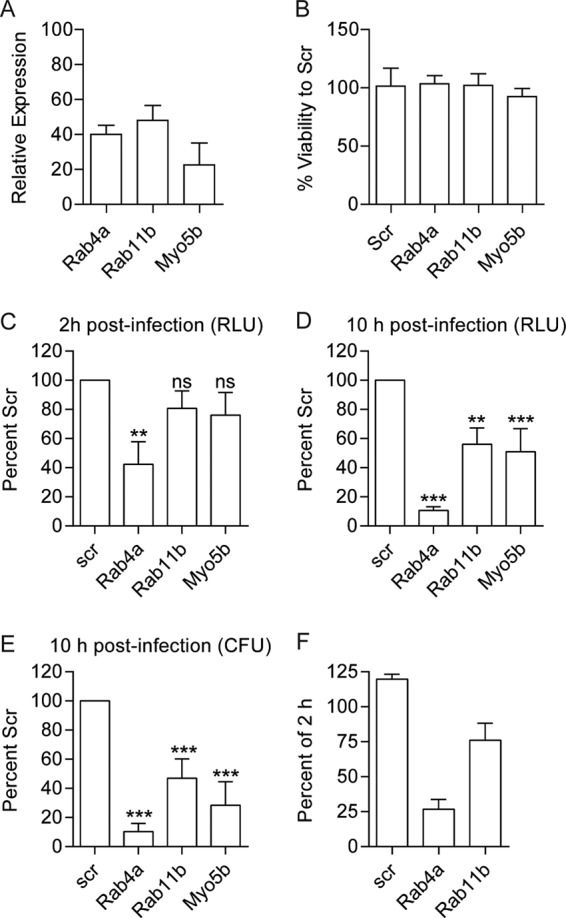
Rab4a, Rab11b, and Myo5b are required for intracellular survival of *Y. pestis*. RAW 264.7 macrophages were transfected with siRNAs targeting Rab4a, Rab11b, or Myo5B. (A and B) Forty-eight hours after transfection, RNA samples were harvested for qRT-PCR (*n* = 9) (represented as relative expression of scrambled-siRNA-treated cells) (A) or cell viability was determined (*n* = 5) (B). (C and D) To determine the impact of RNAi on *Y. pestis* survival, transfected RAW264.7 macrophages (*n* = 6) were infected with *Y. pestis* CO92 pCD1^(-)^ Lux_PtolC_ (MOI of 10), and intracellular bacterial numbers were determined by bioluminescence (in RLU) at 2 h (C) or 10 h (D) postinfection and compared to the values for scrambled (Scr) controls. (E) At 10 h postinfection, a subset of samples (*n* = 3) were harvested for conventional bacterial enumeration. (F) Percent of intracellular bioluminescence at 10 h postinfection compared to 2 h postinfection. Values that are significantly different by one-way ANOVA with Tukey’s posthoc test are indicated by asterisks as follows: **, *P* ≤ 0.01; ***, *P* ≤ 0.001. Values that are not significantly different (ns) are indicated.

Rab4a, Rab11b, and Myo5b are well-characterized contributors to cell recycling (53). While Rab4a was a validated gene in our screen and interconnected to the largest enriched ontology (vesicle-mediated trafficking), the other two genes did not pass the primary screen criteria. RNAi of Rab11b inhibited *Y. pestis* survival by only 50% in the primary screen, and Myo5B was cytotoxic (upon subsequent analysis, only one of the three Myo5B siRNAs used in the primary screen was cytotoxic; this siRNA was not included in further studies). However, because of the importance of these proteins in the recycling pathway, we chose to independently verify the contributions of Rab4a, Rab11b, and Myo5b to the intracellular survival of *Y. pestis* (Fig. 3 and Fig. S1). RNAi resulted in >50% knockdown of each gene target (Fig. 3A) with no significant loss in cell viability (Fig. 3B). Subsequent infection confirmed that knockdown of all three genes impacted the ability of *Y. pestis* to survive within the macrophage. Knockdown of Rab4a had the largest impact, inhibiting *Y. pestis* survival by >40% at 2 h (Fig. 3C; *P* < 0.001) and >80% at 10 h (Fig. 3D; *P* < 0.001). Interestingly, knockdown of Rab11b and Myo5B had no significant impact on *Y. pestis* survival at 2 h (Fig. 3C) but attenuated *Y. pestis* by >40% at 10 h (Fig. 3D; *P* < 0.001). Bioluminescence data were confirmed at 10 h by conventional bacterial enumeration (Fig. 3E). Furthermore, while RNAi of Rab11b had a minor impact on intracellular survival between 2 h and 10 h postinfection, RNAi of Rab4a had a greater impact on the ability of bacteria to survive intracellularly between these two time points (Fig. 3F). Importantly, knockdown of Rab4a or Rab11b did not alter the expression of Rab GTPases involved with phagolysosome maturation or impact phagocytosis of *Y. pestis* (Fig. S2). Together, these data indicate that *Y. pestis* requires the host cell recycling pathway to avoid killing by macrophages.

10.1128/mBio.01800-17.1 FIG S1  RNAi targeting Rab4a, Rab11b, or Myo5b inhibits intracellular survival of *Y. pestis*. RAW264.7 macrophages (*n* = 4) were reverse transfected with three unique siRNAs (represented as 1, 2, and 3) targeting Rab4A, Rab11b, or Myo5b. Forty-eight hours after transfection, macrophages were infected with *Y. pestis* CO92 pCD1^(-)^ Lux_PtolC_ (MOI of 10), and intracellular bacterial numbers were determined by bioluminescence (in RLU) at 10 h postinfection and compared to the values for the scrambled (scr) siRNA control. Values that are significantly different from the values for the scrambled siRNA control by one-way ANOVA with Dunnett’s posthoc test are shown by asterisks as follows: ****, *P* ≤ 0.0001; **, *P* ≤ 0.01. Download FIG S1, TIF file, 0.2 MB. Copyright © 2018 Connor et al. 2018 Connor et al. This content is distributed under the terms of the Creative Commons Attribution 4.0 International license.

10.1128/mBio.01800-17.2 FIG S2  RNAi of Rab4a and Rab11b does not impact expression of other Rab genes or phagocytosis of *Y. pestis*. RAW264.7 macrophages (*n* = 9) were reverse transfected with Rab4a (A) or Rab11b (B) siRNAs, and relative expression of the genes indicated were determined by qRT-PCR. Expression was normalized to GAPDH transcript (Δ*C*_*T*_) and compared to control cells transfected with scrambled siRNA (control [CTR]). Values that are not significantly different (ns) by Student’s *t* test are indicated. (C) RAW264.7 macrophages were transfected with the indicated siRNAs and infected with *Y. pestis* CO92 pCD1^(-)^ pGEN222::GFP. At 20 min postinfection, cells were fixed with 2.5% PFA and blocked with 3% BSA overnight. Extracellular bacteria were specifically labeled with rabbit anti-*Y. pestis* serum (1:1,000) and anti-rabbit Alexa Fluor 594 antibody without cell permeabilization, and extracellular and intracellular bacterial numbers were determined by confocal microscopy as described previously (35). One-way ANOVA with Dunnett’s posthoc test was performed, and the results were compared to control Scr values and indicated as follows: ns, not significant; **, *P* ≤ 0.01. Download FIG S2, TIF file, 1.6 MB. Copyright © 2018 Connor et al. 2018 Connor et al. This content is distributed under the terms of the Creative Commons Attribution 4.0 International license.

### *Y. pestis* requires Rab4a to avoid YCV acidification and lysosomal fusion.

**FIG 4:**
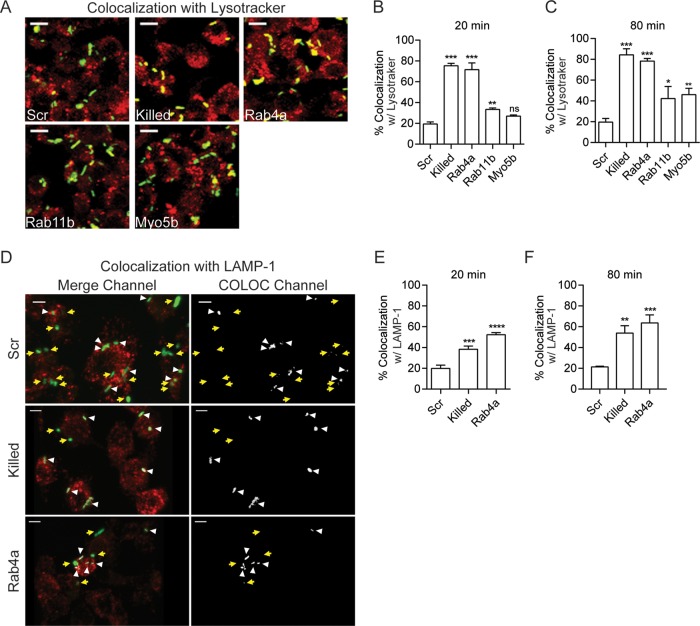
Rab4a is essential for *Y. pestis* to avoid phagosome acidification and LAMP-1 acquisition. (A) Representative confocal microscopy images of RAW264.7 macrophages transfected with the indicated siRNAs that were treated with Lysotracker and infected with *Y. pestis* CO92 pCD1^(-)^ pGEN222, which expresses EGFP (MOI of 7.5). The different colors indicate the following: Lysotracker (red), *Y. pestis* (green), and colocalization (yellow). Bars = 10 µm. (B and C) *Yersinia*-containing vacuole (YCV) colocalization with Lysotracker was calculated using IMARIS at 20 min (B) and 80 min (C) postinfection. (D) Representative confocal microscopy images of RAW264.7 macrophages transfected with scrambled or Rab4a siRNAs that were infected with *Y. pestis* CO92 pCD1^(-)^ pGEN222 (MOI of 3) and stained with anti-LAMP-1 antibody. LAMP-1 is shown in red, and *Y. pestis* is shown in green. Yellow arrows indicate locations of bacteria that do not colocalize with LAMP-1 and white arrowheads indicate bacteria that colocalize with LAMP-1 based on Imaris COLOC function. Bars = 5 µm. (E and F) YCV colocalization with LAMP-1 was calculated using IMARIS at 20 min (E) and 80 min (F) postinfection. One-way ANOVA with Tukey’s posthoc test was performed, and the results are indicated as follows: ns, not significant; *, *P* ≤ 0.05; **, *P* ≤ 0.01; ***, *P* ≤ 0.001. The treatments are indicated as follows: Scr, scrambled siRNA; Killed, untransfected macrophages infected with PFA-killed *Y. pestis* CO92 pCD1^(-)^ pGEN222; Rab4a, Rab4a siRNA; Rab11b, Rab11b siRNA; Myo5b, Myo5b siRNA.

We previously showed that RNAi of Rab1b significantly reduces intracellular survival of *Y. pestis* at 2 h postinfection, which directly correlated with an increase in the frequency of YCV acidification (35). Therefore, we next determined the impact of Rab4a, Rab11b, and Myo5b RNAi on YCV acidification using Lysotracker (Fig. 4A to C). As previously shown (27, 35), *Y. pestis* actively inhibited YCV acidification, with only ~20% of YCVs containing live *Y. pestis* colocalizing with Lysotracker by 80 min postinfection. In contrast, YCVs containing paraformaldehyde (PFA)-killed *Y. pestis* rapidly acidified, with >75% colocalization by 20 min postinfection. As predicted by our relative light unit (RLU) data, Rab4a knockdown resulted in a significant increase in the frequency of YCV acidification (*P* ≤ 0.001), approaching levels similar to PFA-killed bacteria. Unlike Rab4a, RNAi of Rab11b and Myo5b resulted in only a slight increase in colocalization, and by 80 min, colocalization remained significantly lower than both Rab4a siRNA-treated cells or cells infected with PFA-killed *Y. pestis*.

Phagosome acidification is directly linked to lysosomal fusion (57), so we next asked whether Rab4a is required for *Y. pestis* to avoid YCV fusion with lysosomal compartments by measuring YCV colocalization with the LAMP-1 lysosomal marker in siRNA-treated macrophages (Fig. 4D to F). As observed for YCV acidification, *Y. pestis* actively inhibits lysosomal fusion, with only ~20% of YCVs colocalizing with LAMP-1 during the first 80 min of infection. In contrast, YCVs containing PFA-killed *Y. pestis* reached >50% colocalization with LAMP-1 by this time point (*P* ≤ 0.01). Knockdown of Rab4a also inhibited the ability of *Y. pestis* to avoid lysosomal fusion, resulting in a significant increase in colocalization with LAMP-1 (63.7%; *P* ≤ 0.001). Together, these data suggest that Rab4a is required for *Y. pestis* to avoid phagosomal acidification and lysosomal fusion.

### The *Yersinia*-containing vacuole acquires recycling endosome markers.

**FIG 5:**
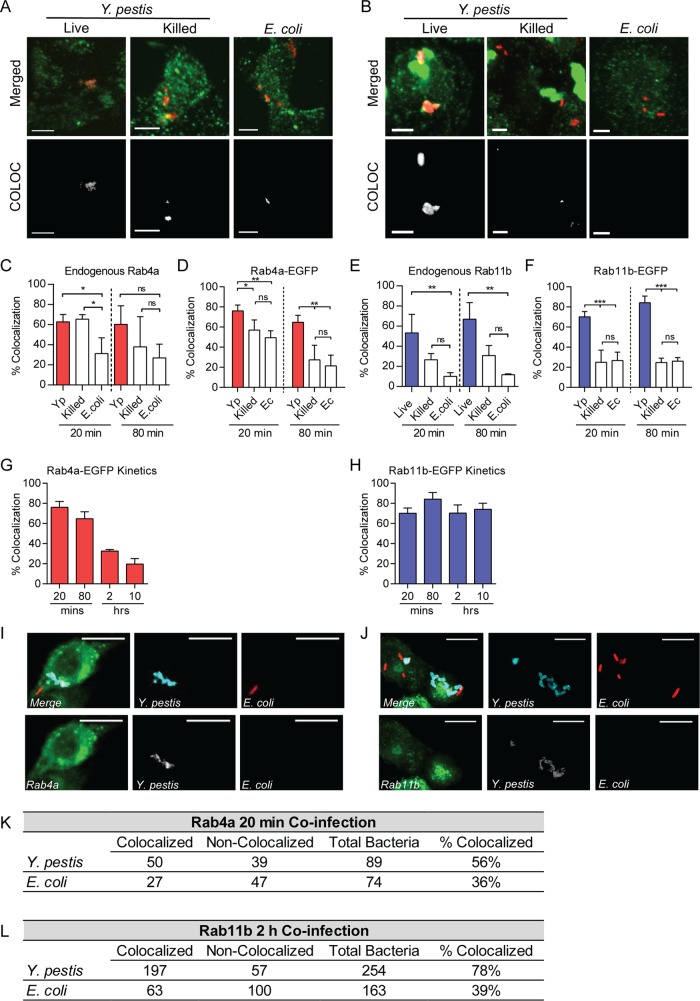
*Y*. *pestis* recruits Rab4a and Rab11b to the YCV. (A and B) Representative confocal microscopy images of primary peritoneal macrophages infected with *Y. pestis* CO92 pCD1^(-)^ pGEN::mCherry (Live) (MOI of 3), PFA-killed *Y. pestis* CO92 pCD1^(-)^ pGen::mCherry (Killed) (MOI of 3), or *E. coli* K-12 pGEN::mCherry (*E. coli*) (MOI of 20) and labeled with anti-Rab4a (A) or anti-Rab11b (B) antibodies. Merged (bacteria [red] and Rab protein [green]) and YCV-Rab colocalization fields generated by Imaris (COLOC) are shown. Bars = 5 µm. (C) Frequency of colocalization of bacterium-containing vacuoles with endogenous Rab4a in peritoneal macrophages. (D) Frequency of colocalization of bacterium-containing vacuoles with Rab4a-EGFP in RAW264.7 macrophages transfected with pEGFP-Rab4a. Yp, *Y. pestis*; Ec, *E*. *coli*. (E) Frequency of colocalization of bacterium-containing vacuoles with endogenous Rab11b in peritoneal macrophages. (F) Frequency of colocalization of bacterium-containing vacuoles with Rab11b-EGFP in RAW264.7 macrophages transfected with pEGFP-Rab11b. (G) Frequency of YCV colocalization with Rab4a-EGFP during 10 h of *Y. pestis* CO92 pCD1^(-)^ pGEN::mCherry infection of RAW264.7 macrophages transfected with pEGFP-Rab4a. (H) Frequency of YCV colocalization with Rab11b-EGFP during 10 h of *Y. pestis* CO92 pCD1^(-)^ pGEN::mCherry infection of RAW264.7 macrophages transfected with pEGFP-Rab11b. (I and J) Representative images of RAW264.7 macrophages transiently transfected with pEGFP-Rab4a (I) or pEGFP-Rab11b (J) and coinfected with *Y. pestis* CO92 pCD1^(-)^ (blue) (MOI of 3) or *E. coli* K-12 pGEN::mCherry (red) (MOI of 20). Bars = 10 µm. (K and L) Frequency of colocalization of *Y. pestis-* or *E. coli*-containing vacuoles in coinfected cells withEGFP-Rab4a (K) or EGFP-Rab11b (L). One-way ANOVA with Tukey’s posthoc test was performed, and the results are indicated as follows: ns, not significant; *, *P* ≤ 0.05; **, *P* ≤ 0.01; ***, *P* ≤ 0.001.

To determine whether Rab4a or Rab11b is recruited to the YCV, primary peritoneal macrophages were infected with *Y. pestis*, PFA-killed *Y. pestis*, or *Escherichia coli* K-12, and recruitment of endogenous Rab4a and Rab11b to the YCV was determined by immunofluorescence using anti-Rab antibodies (Fig. 5A to F). At 20 min postinfection, a significantly higher number of YCVs colocalized with Rab4a and Rab11b than vacuoles containing *E. coli* (Fig. 5C and E; *P* ≤ 0.05 and *P* ≤ 0.01, respectively). By 80 min postinfection, YCVs retained significantly higher colocalization with Rab11b than *E. coli*-containing vacuoles (*P* ≤ 0.01). However, while YCVs trended toward higher colocalization with Rab4A compared to vacuoles containing *E. coli* or killed *Y. pestis*, these differences were not statistically different. To confirm these results, RAW264.7 macrophages were transfected with plasmids expressing Rab4a or Rab11b fused to enhanced green fluorescent protein (EGFP) to monitor the localization of Rab proteins independently of antibodies (58) and 24 h later infected with bacteria. At 20 min postinfection, ~50% of PFA-killed *Y. pestis* and *E. coli* K-12 containing phagosomes colocalized with Rab4a-EGFP (Fig. 5D). However, as observed in primary macrophages, a significantly higher number of vacuoles containing live *Y. pestis* colocalized with Rab4a-EGFP (76%; *P* ≤ 0.01). By 80 min postinfection, both PFA-killed *Y. pestis* and *E. coli* K-12 decreased in colocalization with Rab4a-EGFP, indicating loss of the GTPase during phagosome maturation, but vacuoles containing live *Y. pestis* maintained Rab4a-EGFP at a statistically higher frequency (~61%; *P* ≤ 0.01). As the infection continued, colocalization of the YCV with Rab4a-EGFP decreased, and by 2 and 20 h postinfection, it approached background levels (Fig. 5G). In contrast to Rab4a-EGFP, fewer vacuoles containing PFA-killed *Y. pestis* or *E. coli* K-12 colocalized with Rab11b-EGFP at 20 and 80 min postinfection (Fig. 5F). However, the majority of YCVs containing live *Y. pestis* colocalized with Rab11b-EGFP, approaching 85% by 80 min postinfection (*P* ≤ 0.001). Furthermore, *Y. pestis* continued to colocalize with Rab11b-EGFP at a high frequency throughout the course of the infection (Fig. 5H).

Finally, to determine whether Rab proteins are specifically recruited to phagosomes containing *Y. pestis* and not to all phagosomes of *Y. pestis*-infected macrophages, RAW264.7 macrophages transfected with Rab4a-EGFP or Rab11b-EGFP were coinfected with *Y. pestis* and *E. coli* K-12. Colocalization between Rab4a-EGFP and bacterium-containing vacuoles at 20 min postinfection and between Rab11b-EGFP and bacterium-containing vacuoles at 2 h postinfection was determined by confocal microscopy (Fig. 5I to L). In cells infected with both bacteria, *Y. pestis*-containing vacuoles had a higher frequency of colocalization with both Rab proteins than *E. coli* K-12-containing vacuoles. Furthermore, the frequency of *E. coli* K-12 colocalization was not higher during coinfection than observed during single infection with just *E. coli* K-12 (Fig. 5D and F). Together, these data indicate that *Y. pestis* actively recruits Rab4a and Rab11b to the YCV during early stages of macrophage infection and that recruitment is specifically to vacuoles containing live *Y. pestis*.

### *Y. pestis* infection disrupts host recycling.

**FIG 6:**
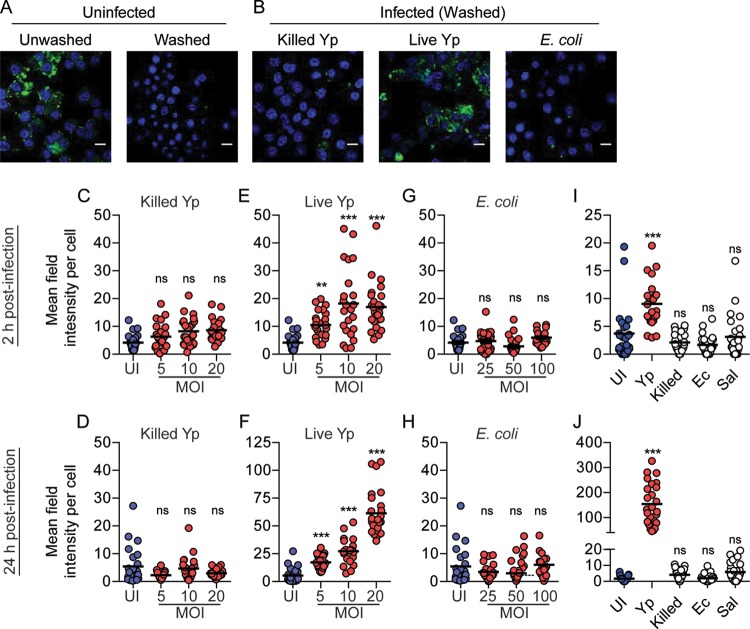
*Y*. *pestis* infection disrupts host cell recycling. (A) Representative images of uninfected RAW264.7 macrophages showing total TfR (Unwashed) and intracellular TfR remaining after washing with high-salt, low-pH buffer to remove antibody labeling of extracellular receptors (Washed). (B) Representative images of uninfected RAW264.7 macrophages infected with *Y. pestis* CO92 pCD1^(-)^ (Live Yp) (MOI of 20), PFA-killed *Y. pestis* CO92 pCD1^(-)^ (Killed Yp) (MOI of 20), or *E. coli* K-12 (MOI of 100) for 24 h and then washed with high-salt, low-pH buffer to remove antibody labeling of extracellular receptors. The nuclei are stained with DAPI (blue) and TfR (green). Bars = 10 µm. (C to H) Mean field intensity of TfR signal per cell was calculated by confocal microscopy at 2 and 24 h postinfection with PFA-killed *Y. pestis* CO92 pCD1^(-)^ (Killed Yp) (C and D), live *Y. pestis* CO92 pCD1^(-)^ (Live Yp) (E and F), or *E. coli* K-12 (G and H). UI, uninfected. (I and J) Mean field intensity of TfR signal per cell from hMDMs infected with live *Y. pestis* CO92 pCD1^(-)^ (Yp) (MOI of 10), PFA-killed *Y. pestis* CO92 pCD1^(-)^ (Killed) (MOI of 10), *E. coli* K-12 (Ec) (MOI of 100), or *S. enterica* Typhimurium (Sal) (MOI of 100) for 2 h (I) or 24 h (J). Data from one experiment representative of three independent experiments are shown. Each data point represents the mean field intensity (TfR) per cell from an individual field (*n* = 25; ~100 cells per field). The bars represent the means. One-way ANOVA with Dunnett’s posthoc test was performed (values compared to uninfected values), and the results are shown as follows: ns, not significant; **, *P* ≤ 0.01; ***, *P* ≤ 0.001.

Recruitment of Rab4a and Rab11b to the YCV indicated that *Y. pestis* remodels its phagosome to resemble a recycling endosome. Because of these links to host cell recycling, we next tested whether infection with *Y. pestis* impacts host cell recycling by monitoring recycling of the host transferrin receptor (TfR). RAW264.7 macrophages were infected with *Y. pestis* CO92, PFA-killed *Y. pestis*, or *E. coli* K-12. At 2 and 24 h postinfection, intracellular TfRs were differentially labeled from extracellular TfRs, and intracellular TfR intensity was determined by microscopy (Fig. 6A and B). Unlike PFA-killed bacteria, infection with live *Y. pestis* significantly disrupted recycling of TfR as early as 2 h postinfection, resulting in accumulation of intracellular TfR, and continued to impact recycling for 24 h (Fig. 6C to F). Furthermore, intracellular TfR intensity also increased as the *Y. pestis* multiplicity of infection (MOI) increased. Inhibition of TfR recycling was also specific for macrophages containing intracellular *Y. pestis*, as uninfected cells from the same cultures did not show elevated retention of TfR (Fig. S5). Importantly, infection with 10-fold-higher numbers of *E. coli* K-12 did not result in a significant change in TfR retention compared to uninfected macrophages (Fig. 6G and H), indicating that recycling disruption is not a general response to macrophage activation.

Next, we confirmed that disruption of recycling occurs during infection of primary human monocyte-derived macrophages (hMDMs). As observed for RAW264.7 macrophages, infection of hMDMs with *Y. pestis* resulted in a significant increase in intracellular TfR intensity, while infection with PFA-killed *Y. pestis* or *E. coli* K-12 had no significant impact on recycling (Fig. 6I and J). In contrast to *Y. pestis*, infection with *Salmonella enterica* serotype Typhimurium, another intracellular pathogen, had no significant impact on TfR recycling (Fig. 6I and J) (*S. enterica* Typhimurium infection of macrophages was independently monitored to ensure bacterial infection, growth, and host cell viability [Fig. S3]). Importantly, intracellular growth of *Y. pestis* was not impacted by incubation with TfR antibody (Fig. S4). Together, these data demonstrate that *Y. pestis* actively disrupts host cell recycling, disruption is not a default response by macrophages to bacteria, and disruption is pathogen specific.

10.1128/mBio.01800-17.3 FIG S3  Survival of *S. enterica* Typhimurium in macrophages. (A) Serial dilutions of *S. enterica* Typhimurium pGEN–Lux (*n* = 3) were analyzed using a plate reader to determine bioluminescence (in RLU) and plated on agar to determine CFU. RLU and CFU were compared to determine whether there was a direct correlation between the two (*R*^2^). The limit of detection (LD) was also calculated. (B) To determine the survival of *S. enterica* Typhimurium in macrophages, RAW264.7 macrophages were infected with *S. enterica* Typhimurium pGEN–Lux at an MOI of 10 or 100. One hour postinfection, extracellular bacteria were killed with 100 µg/ml gentamicin for 1 h, and then the medium was replaced with 10 µg/ml gentamicin to maintain intracellular bacteria for 24 h. Intracellular survival as indicated by bioluminescence was monitored every 2 h for 24 h. (C) To determine whether human-derived monocytes (hMDMs) were killed by *S. enterica* Typhimurium, hMDMs were infected with *S. enterica* Typhimurium pGEN222-P_*Em7*_::DsRED (MOI of 100) for 24 h. Cells were stained with DAPI, and nuclei were quantified by confocal microscopy as an indicator of intact host cells (*n* = 50). pV, *P* value. Download FIG S3, TIF file, 1.2 MB. Copyright © 2018 Connor et al. 2018 Connor et al. This content is distributed under the terms of the Creative Commons Attribution 4.0 International license.

10.1128/mBio.01800-17.4 FIG S4  Anti-TfR antibody does not alter *Y. pestis* phagocytosis or intracellular growth. RAW264.7 macrophages were incubated with medium containing anti-TfR antibody and infected with *Y. pestis* CO92 pCD1^(-)^ Lux_PtolC_ at the indicated MOIs (*n* = 8). Twenty minutes postinfection, extracellular bacteria were killed with gentamicin for 1 h, and the medium was replaced with fresh medium containing gentamicin and anti-TfR antibody. Intracellular bacterial numbers were monitored by bioluminescence (in RLU) at indicated time points over a 24-h period. Download FIG S4, TIF file, 0.7 MB. Copyright © 2018 Connor et al. 2018 Connor et al. This content is distributed under the terms of the Creative Commons Attribution 4.0 International license.

10.1128/mBio.01800-17.5 FIG S5  TfR recycling is only inhibited in macrophages directly infected with *Y. pestis*. RAW264.7 macrophages were incubated with anti-TfR antibody and infected with *Y. pestis* CO92 pCD1^(-)^ (MOI of 20) for 24 h. Cells were washed with a high-salt, low-pH buffer to remove antibody labeling of extracellular receptors, then permeabilized, and incubated with secondary antibody. Cells were imaged by confocal microscopy, and uninfected (UI) and infected (I) cells were manually isolated using IMARIS to maintain host cell boundaries and analyzed for intracellular TfR intensity. Data from one experiment representative of three independent experiments are shown. Each symbol represents the value for a single cell (*n* = 50). The red bars represent the mean values. Paired Student’s *t* test was used to compare samples (***, *P* ≤ 0.001). Download FIG S5, TIF file, 0.8 MB. Copyright © 2018 Connor et al. 2018 Connor et al. This content is distributed under the terms of the Creative Commons Attribution 4.0 International license.

### Disruption of host cell recycling is required for *Y. pestis* replication.

**FIG 7:**
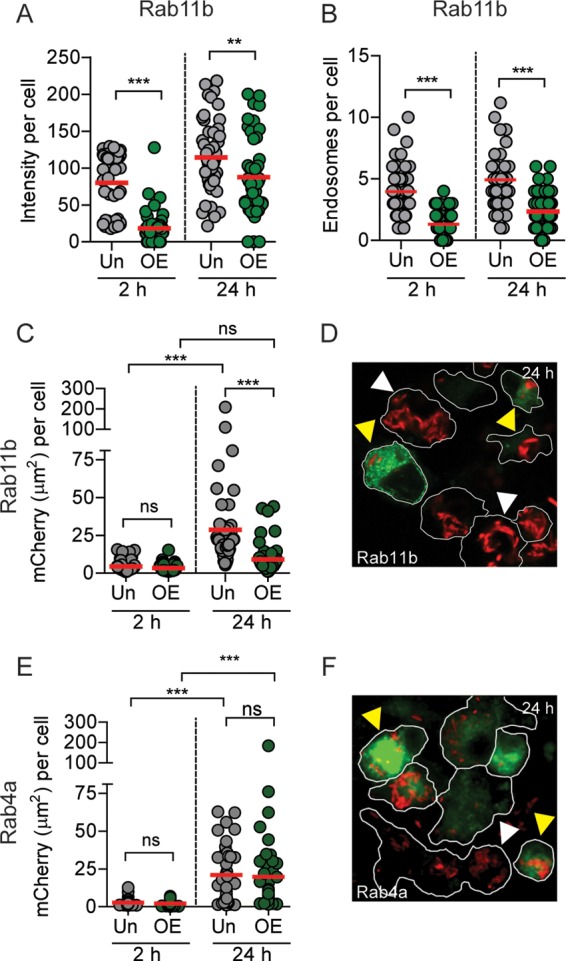
Overexpression of Rab11b restores recycling and inhibits intracellular growth of *Y. pestis*. (A to D) RAW264.7 macrophages were transiently transfected with pEGFP-Rab11b prior to infection with *Y. pestis* CO92 pCD1^(-)^ pGEN::mCherry (MOI of 10), and TfR was differentially labeled and imaged by confocal microscopy. Individual Rab11b-overexpressing transfected (OE) and untransfected (Un) cells were manually isolated using IMARIS to maintain host cell boundaries and analyzed for intracellular TfR and bacteria (*n* = 50 cells each). (A) Intracellular TfR intensity per cell. (B) Number of TfR-positive endosomes per cell. (C) Intracellular bacterial numbers as a function of mCherry signal area per cell. (D) Representative image of infected cells overexpressing Rab11b at 24 h postinfection. (E) Intracellular bacterial numbers as a function of mCherry signal area per cell for cells overexpressing Rab4a (OE) compared to untransfected cells (Un). (F) Representative image of infected cells from Rab4a overexpression studies at 24 h postinfection. Data from one experiment representative of three independent experiments are shown. Paired Student’s *t* test was used to compare samples from the same time point and ANOVA with Tukey’s posthoc test was used for comparisons different time points (in panels C and E only), and results are indicated as follows: ns, not significant; **, *P* ≤ 0.01; ***, *P* ≤ 0.001. Red bars show the mean values. For microscopy images, *Y. pestis* (red), Rab protein (green), bacteria in untransfected cells (white arrowheads), and bacteria in Rab-overexpressing cells (yellow arrowheads) are indicated. Cell borders are shown outlined in white.

Since Rab11b is recruited to and retained on the YCV, we next explored whether *Y. pestis* infection disrupts host cell recycling through sequestration of Rab11b and depletion of available cellular Rab11b for vesicular trafficking. If this was occurring, then overexpression of Rab11b may be able to restore depleted Rab11b levels and host cell recycling. To test this hypothesis, RAW264.7 macrophages were transfected with a plasmid overexpressing wild-type Rab11b-EGFP (58), infected with *Y. pestis*, and TfR recycling was monitored. Importantly, overexpression of Rab11b-EGFP did not alter TfR recycling in uninfected cells (Fig. S6). At both 2 and 24 h postinfection, intracellular TfR intensity and TfR-positive endosomes per cell were significantly lower in cells overexpressing Rab11b-EGFP (Fig. 7A and B), demonstrating that overexpression of Rab11b restored host cell recycling during infection. To determine whether restoration of recycling impacted intracellular survival of *Y. pestis*, we quantified bacterial numbers as a function of bacterial fluorescence (Fig. 7C and D). At 2 h postinfection, there were no differences in the bacterial numbers of Rab11b-overexpressing and untransfected cells. However, by 24 h postinfection, bacterial numbers significantly increased in the untransfected cells, while the signal area did not increase in transfected cells (*P* ≤ 0.001). Furthermore, overexpression of Rab4a-EGFP, which is not sequestered to the YCV (Fig. 5G), did not alter bacterial replication (Fig. 7E and F). Together, these data suggest that *Y. pestis* infection limits Rab11b availability through sequestration to the YCV, resulting in disruption of host cell recycling. Furthermore, disruption of recycling is required in order for *Y. pestis* to replicate in macrophages.

10.1128/mBio.01800-17.6 FIG S6  Overexpression of Rab11b does not impact TfR recycling. RAW264.7 macrophages were transiently transfected with pEGFP-Rab11b. Twenty-four hours after transfection, cells were incubated with anti-TfR for 2 h. Cells were washed with high-salt, low-pH buffer to remove antibody labeling of extracellular receptors, then permeabilized, and incubated with secondary antibody. Cells were imaged using confocal microscopy, and individual Rab11b-overexpressing transfected (OE) and untransfected (Un) cells were manually isolated using IMARIS to maintain host cell boundaries and analyzed for intracellular TfR intensity. Data from one experiment representative of three independent experiments are shown. Each symbol represents the value for a single cell (*n* = 50). The red bars represent the mean values. Paired Student’s *t* test was used to compare samples, and no significant differences were observed. Download FIG S6, TIF file, 0.7 MB. Copyright © 2018 Connor et al. 2018 Connor et al. This content is distributed under the terms of the Creative Commons Attribution 4.0 International license.

## DISCUSSION

**FIG 8:**
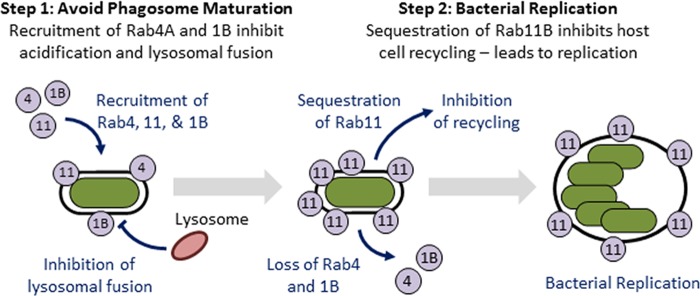
Model of the biogenesis of the *Yersinia*-containing vacuole (YCV). *Y. pestis* engages the host endosome recycling pathway by recruiting Rab GTPases to the YCV in order to generate a protective replicative niche in a two-step process. First, Rab1b and Rab4a are recruited to the YCV, which is required for the bacterium to inhibit phagosome acidification and fusion with the lysosome. While these two Rab proteins are eventually lost from the YCV, Rab11b is retained on the YCV over the entire course of infection. Retention of Rab11b leads to a global inhibition of host recycling, which is required for *Y. pestis* to replicate in macrophages. Rab4, Rab11, and Rab1b proteins are shown in the figure as gray circles labeled 4, 11, and 1B, respectively.

While it has been known for decades that *Y. pestis* survives within a vacuolar compartment within macrophages (27, 28), the mechanisms leading to subversion of phagolysosome killing by macrophages have not been defined. To better understand the processes involved in the biogenesis of the protective YCV by *Y. pestis*, we conducted an RNAi genome-wide screen that identified 71 host proteins necessary for survival of *Y. pestis* inside macrophages. Bioinformatic analysis showed enrichment for three key cellular processes: vesicular trafficking, vesicular transport, and vesicular localization. Refining the interactome generated from this screen suggested that the host endocytic recycling pathway is key for *Y. pestis* to survive in macrophages. Expanding on these findings, we demonstrated for the first time that *Y. pestis* remodels the YCV by recruiting endocytic recycling compartment (ERC) markers Rab4a and Rab11b. Importantly, trafficking of endosomes to the ERC, which is mediated by Rab4 and Rab11, is thought to prevent cargo within these compartments from entry into degradative compartments such as phagolysosomes (53). Therefore, by remodeling the YCV, *Y. pestis* may take advantage of this normal, nondegradative pathway to avoid lysosomal fusion and escape killing by the macrophage. A model summarizing additions to the YCV biogenesis process based on these data is outlined in Fig. 8.

While Rab4a and Rab11b are recruited to the YCV, RNAi of these two genes resulted in very distinct phenotypes. Knockdown of Rab4a resulted in rapid killing of *Y. pestis* within 2 h of infection, while changes in bacterial survival in Rab11b siRNA-treated macrophages were not apparent until later. These findings suggest that Rab4a is required to avoid early steps in phagosome maturation, while Rab11b interactions contribute to later stages in YCV biogenesis. Supporting this hypothesis, we observed that Rab4a is required to avoid phagosome acidification and lysosomal fusion, which begins within 20 min of phagocytosis. We have also shown that Rab1b is also required to avoid acidification (35), highlighting that avoidance of YCV acidification is a key step in the intracellular survival of *Y. pestis*. Future studies to better understand the kinetics of Rab1b, Rab4a, and Rab11b recruitment and retention will help understand the dynamics of these early steps in YCV biogenesis and whether these proteins are dependent on one another for stepwise recruitment. Importantly, direct recruitment of Rab1b and Rab4a to the YCV in order to inhibit acidification could also explain why live *Y. pestis* did not inhibit the acidification of phagosomes containing PFA-killed *Y. pestis* in coinfected macrophages previously reported by Pujol et al. (27). This is further supported by our data here that live bacteria recruit Rab4a and Rab11b only to the YCV they are contained within (Fig. 5K and L). Furthermore, these data also suggest the bacterial factors that mediate Rab recruitment are likely located at the YCV and not distributed throughout the cell.

In contrast to Rab1b and Rab4a, knockdown of Rab11b had only a minor impact on YCV acidification, with the majority of YCVs still avoiding acidification (58% versus 22% for Rab4A RNAi at 80 min). This change in YCV acidification frequency correlated with increased bacterial survival at 2 h postinfection in Rab11b siRNA-treated cells (Fig. 3C). However, we observed significantly lower bacterial numbers in Rab11b siRNA-treated macrophages at 10 h postinfection. Notably, while the number of *Y. pestis* in Rab11b siRNA-treated macrophages decreased slightly between 2 and 10 h postinfection, bacteria were beginning to replicate in the scrambled-siRNA-treated cells (Fig. 3F). These data indicate that Rab11b knockdown may restrict intracellular replication of *Y. pestis* as opposed to survival. *Anaplasma phagocytophilum* (59) and *Chlamydia* species (60) have also been shown to recruit Rab4 and Rab11 to their vacuoles, and knockdown of Rab11 in cells infected with *Chlamydia trachomatis* (61) and *Coxiella burnetii* (39) inhibits bacterial replication of these species. During infection, these three pathogens develop large vacuoles, which require acquisition of vesicular membranes. It has been suggested that interception of Rab11-positive recycling endosomes could provide both membranes for vacuole expansion and nutrients for bacterial replication (59, 61). The YCV also expands into a spacious vacuole after ~8 h of infection, though vacuoles do not expand in size to the degree observed for the aforementioned pathogens (16, 27, 28). This time frame coincides with the beginning of intracellular replication of *Y. pestis*, suggesting that *Y. pestis* may also intercept recycling endosomes for similar purposes. During these studies, we did not observe evidence for the formation of spacious YCVs in Rab11b siRNA-treated macrophages, supporting that hijacking of recycling endosomes may be occurring during *Y. pestis* infection and suggesting convergent evolution by multiple intracellular pathogens. However, if direct interception of recycling endosomes is the only function of Rab11b targeting during *Y. pestis* infection, we would not expect that overexpression of Rab11b would inhibit bacterial growth, as the YCV still acquires Rab11b in overexpressed cells and should still be able to intercept recycling endosomes. Importantly, while Huang et al. and Rzomp et al. used overexpression of Rab-GFPs to localize the Rab4 and Rab11 to the *Anaplasma*- and *Chlamydia*-containing vacuoles, they did not report inhibition in bacterial growth during overexpression of Rab11 (59, 60). These data indicate that inhibition of intracellular replication is not a general artifact of Rab11 overexpression, but perhaps a pathogen-specific phenotype. Therefore, while our current data do not exclude interception of recycling endosomes through Rab11b, our overexpression data suggest that a novel mechanism used by *Y. pestis* to manipulate the biology of the macrophage, which is yet to be defined, contributes to intracellular replication of *Y. pestis*.

We also demonstrated for the first time that macrophage recycling is disrupted during *Y. pestis* infection. This disruption does not appear to be a general response of macrophages to encountering bacteria, as infection with PFA-killed *Y. pestis*, *E. coli* K-12, or *S. enterica* Typhimurium did not disrupt recycling in macrophages. While other bacterial pathogens have been shown to recruit recycling markers to their vacuole, to our knowledge, this is the first description of a bacterial infection disrupting global endosome recycling during infection. Disruption of host recycling by *Y. pestis* could alter several different aspects of macrophage biology that could impact infection. For example, Longatti et al. have shown that disruption of recycling inhibits starvation-induced autophagy and autophagy induction is dependent on active Rab11 (62, 63). More recently, Szatmári et al. demonstrated that Rab11b interacts with Hook, a negative regulator of endosome maturation, to facilitate cross talk between recycling endosomes and induction of autophagy (64). These links between Rab11, recycling, and autophagy could be important in the context of *Y. pestis* infection. Several studies have shown that both *Y. pestis* and *Yersinia pseudotuberculosis* induce autophagy during infection, and eventually YCVs take on characteristics of autophagosomes (65). Interestingly, autophagy does not seem to be detrimental to *Y. pestis* (27), but it may be required for bacterial replication (66). Furthermore, *Y. pestis* does not appear to escape the YCV, raising questions of why is autophagy triggered and how is the YCV targeted for autophagosome formation. Our discovery that *Y. pestis* interacts with Rab11b and the host recycling pathway provide potential answers to these questions. For example, disruption of endocytic recycling may induce conditions/signals similar to starvation that trigger autophagy pathways in the macrophage. Additionally, sequestration of Rab11b by *Y. pestis* may trigger autophagy nucleation at the YCV, resulting in autophagosome formation. Therefore, the consequence of inhibiting host cell recycling by *Y. pestis* may be to specifically induce autophagy to promote replication. Future studies to address these hypotheses are ongoing and important to define the contribution of Rab11b sequestration and recycling disruption on the pathogenesis of *Y. pestis*.

In summary, we have demonstrated for the first time that *Y. pestis* remodels the YCV to resemble a recycling endocytic vacuole in a T3SS-independent manner, and the bacterium disrupts host recycling during infection. These findings suggest that interactions with the recycling pathway are important for multiple steps in the YCV biogenesis process. We also showed that overexpression of Rab11b overcomes the ability of *Y. pestis* to disrupt recycling and this prevents bacterial replication, suggesting that recruitment of Rab11b to the YCV has *trans*-acting effects on the cell that are beneficial to the bacterium. Future studies to further define the impact disruption of host cell recycling has on the biology of the macrophage will be important to understand how *Y. pestis* avoids killing by these phagocytes.

## MATERIALS AND METHODS

### Eukaryotic cells, bacterial strains, and plasmids.

RAW264.7 macrophages were obtained from ATCC and cultured in Dulbecco modified Eagle medium (DMEM) containing 100 mM glucose plus 10% fetal bovine serum (FBS) (HyClone). Peritoneal macrophages were isolated from C57BL/6 mice as previously described (67). Human monocyte-derived macrophages (hMDMs) were isolated with minor modifications as previously described (68–70). Briefly, hMDMs were isolated from peripheral blood samples from healthy, antibiotic-free adult donors (institutional review board [IRB] protocol 04.0358) using a Ficoll-Hypaque gradient. Monocytes were suspended in RPMI 1640 plus 20% FBS (Biowest), then aliquoted into six-well ultralow attachment plates (Greiner Bio One), and incubated for 3 days at 37°C and 5% CO_2_. On day 4, nonadherent cells were aspirated, monolayers were scraped and resuspended in RPMI 1640 plus 10% FBS, and 2.5 × 10^5^ cells/well were transferred into the wells of a 24-well tissue culture plate (Greiner Bio One). On days 6 and 7, the medium was removed, and replaced with RPMI 1640 plus 5% FBS and RPMI 1640 plus 1% FBS, respectively. hMDMs were used for studies on day 8.

*Y. pestis* CO92 pCD1^(-)^ (71) and KIMD-19 (BEI Resources NR-4681) were cultivated at 26°C in Difco brain heart infusion broth (Becton, Dickinson, and Co.). *E. coli* K-12 DH5α (New England Biolabs) and *Salmonella enterica* Typhimurium L2 (ATCC 14028s) were cultivated at 37°C in Luria-Bertani broth (Miller) (Becton, Dickinson, and Co.). Bioluminescent derivatives were generated using the Lux_PtolC_ bioreporter as described previously (50). To generate fluorescent bacterial strains, bacteria were transformed with pGEN222, pGEN-P_*EM7*_::DsRED, or pGEN222::mCherry (72) and maintained with 50 µg/ml carbenicillin (Sigma). Inactivation of *Y. pestis* was achieved by incubating bacteria with 2.5% paraformaldehyde (PFA) for 30 min at room temperature as previously described (35). During RAW264.7 macrophage infections, extracellular *Y. pestis* and *E. coli* were killed with a 1-h treatment of 16 µg/ml gentamicin, followed by maintenance in 2 µg/ml; extracellular *S*. Typhimurium was killed with 100 µg/ml gentamicin and maintained in 10 µg/ml. For peritoneal macrophages and hMDMs, the 1-h gentamicin concentration for *Y. pestis* was lowered to 8 µg/ml, with maintenance in 1 µg/ml or no maintenance, respectively (25).

### RNAi and transfection of RAW264.7 macrophages.

To confirm RNAi efficiency in RAW264.7 macrophages, cells were forward transfected with 20 μl of a solution with a final concentration of 1 μM of Silencer siRNA (Life Technologies) diluted in Opti-MEM (Life Technologies) mixed with 10 µl of 0.03% (vol/vol) Lipofectamine RNAiMax/Opti-MEM (Life Technologies) for 48 h (35). Total RNA from 1.6 × 10^6^ transfected cells (*n* = 3) was isolated and converted to cDNA, and quantitative reverse transcription-PCR (qRT-PCR) was performed using Sybr green (Life Technologies) (35). Relative expression was calculated using the ΔΔ*C*_*T*_ method (73). For protein expression, 3 × 10^5^ transfected cells were harvested, and total lysates were analyzed by Western blotting using anti-glyceraldehyde-3-phosphate dehydrogenase (anti-GAPDH) (PA1-988; Pierce), anti-Copβ1 (PA1-061; Pierce), and antiactin (ab8227; Abcam) antibodies as previously described (35). For expression of Rab-EGFP GTPases, RAW264.7 macrophages were transiently transfected with pEGFP-Rab11b and pEGFP-Rab4a (74) using JetPrime as described by the manufacturer (Polyplus).

### RNAi primary and validation screens.

RAW264.7 macrophages were forward transfected with small interfering RNAs (siRNAs) from the Silencer siRNA mouse genome library v3 (Ambion). The library contains three siRNAs targeting each gene, which were pooled for the primary screen. siRNAs were suspended in 20 µl Opti-MEM at a final concentration of 1 µM and then mixed with 10 µl of 0.03% (vol/vol) Lipofectamine RNAiMax/Opti-MEM and added to each well of a white flat-bottom 96-well plate (Greiner Bio One). For each plate, column 12 included wells containing scrambled siRNA (negative control; *n* = 3) and Copβ1 siRNA (positive control; *n* = 3) as controls for transfection efficiency and plate-to-plate variation. The plates were incubated at room temperature for 10 min, and then 1 × 10^4^ RAW264.7 macrophages suspended in 80 µl of DMEM plus 10% FBS (HyClone) were added. The cells were incubated for 48 h and then infected with *Y. pestis* CO92 Lux_PtolC_ pCD1^(-)^ (multiplicity of infection [MOI] of 10) and synchronized by centrifugation (200 × *g*) for 5 min. Twenty minutes after infection, extracellular bacteria were killed with gentamicin as described above. Intracellular bacteria were quantified as a function of bioluminescence at 20 min and 2 h and 10 h postinfection using a Synergy 4 plate reader (BioTek; 1-s read with sensitivity set at 150). After the 10-h bioluminescent read, cell viability was determined using alamarBlue (Life Technologies). Briefly, 10 µl alamarBlue was added directly to each well and incubated at 37°C and 5% CO_2_ for 2 h, and fluorescence was determined using a Synergy 4 plate reader (excitation wavelength, 560 nm; emission wavelength, 600 nm) and compared to the average of the scrambled-siRNA control wells. For each plate, a Z factor (Z′) was calculated from the control wells using the formula: 1 − (3× (SD Copβ1 RLU – SD scrambled RLU)/(AVG scrambled RLU – AVG Copβ1 RLU)) where SD is the standard deviation, AVG is the average. Plates with Z′ of <0.3 were repeated. Intracellular survival was normalized for each plate using the following formula: (siRNA RLU/AVG Copβ1 RLU)/(AVG scrambled RLU/AVG Copβ1 RLU). Primary screen selection criteria was set at ≥60% inhibition of *Y. pestis* survival and ≤50% cytotoxicity. Primary hits were validated using two single siRNAs in independent validation screens. Selection criteria for the validation screen were ≥40% inhibition of *Y. pestis* survival and ≤50% cytotoxicity.

### Bioinformatic analysis.

Validated and primary screen hits were stored with both Entrez Gene and MGI identifiers. Interacting partners were identified using all experimental evidence codes from BioGRID (75) and STRING (76) databases using MGI and Entrez Gene identifiers. Interactors for the input data sets were validated and primary hits were characterized by (i) direct interactions within each individual data set and (ii) direct interactions from validated to primary hits. These interactions were stored and imported into Cytoscape (v3.30) to generate interaction maps (77). For Gene Ontology (GO) clustering, the validated hits were imported by Entrez Gene identifier to Cytoscape plug-ins ClueGO (78) and CluePedia (79). Genes were clustered using all GO evidence codes, a minimum kappa score of 0.4, and a *P* value threshold of 0.05.

### Fluorescence confocal microscopy.

To monitor vacuole acidification, macrophages were incubated with 75 nM Lysotracker red DND-99 for 1 h prior to fixation with 2.5% PFA (35). Endogenous Rab proteins were labeled using anti-Rab4a (10347-1-AP; Protein Tech) and anti-Rab11b (sc-26591; Santa Cruz) antibodies at concentrations of 1:100 and 1:200, respectively. *Y. pestis* was labeled with rabbit anti-*Y. pestis* serum at a 1:10,000 dilution, and the transferrin receptor (TfR) was labeled with anti-TfR antibody (ab84036; Abcam) at 1 µg/ml. All secondary antibodies were obtained from Jackson ImmunoResearch and used at the following concentrations: anti-rabbit antibody conjugated to Alexa Fluor 647 (catalog no. 711-605-152), 1:4,000; anti-goat antibody conjugated to Alexa Fluor 647 (catalog no. 705-605-147), 1:1,000; anti-rabbit antibody conjugated to Alexa Fluor 488 (catalog no. 111-545-144), 1:2,000. Coverslips were mounted with Prolong gold with 4′,6′-diamidino-2-phenylindole (DAPI) for 24 h prior to imaging. All cells were imaged using a Zeiss LSM 710 laser confocal microscope. Colocalization was determined using the COLOC module in IMARIS 8.0 (Bitplane). TfR intensity and mCherry quantification were determined using Fiji (80). To quantify *Y. pestis* replication within cells, individual infected cells were segregated, and the corresponding bacterial fluorescent channel was analyzed for signal area (in square micrometers) per cell using Fiji (80).

### Differential staining of intracellular TfR.

To monitor TfR recycling, macrophages were infected with bacteria as described above in the presence of 1 µg/ml of anti-TfR antibody, which was maintained throughout the course of infection. At 2 and 24 h postinfection, the cells were gently washed three times with ice-cold stripping buffer (Hanks buffered salt solution [HBSS] with 50 mM glycine, 150 mM NaCl, and 0.2% bovine serum albumin [BSA] [pH 4]) to remove anti-TfR antibody bound to the cell surface (extracellular receptors) and nonspecific binding antibody. The cells were then fixed with 2.5% PFA for 15 min at room temperature and incubated with permeabilization buffer (0.5% Tween 20, 3% BSA) overnight at 4°C. For indirect immunofluorescence labeling of internal TfR, cells were incubated for 1 h with anti-rabbit antibody labeled with Alexa Fluor 488 diluted in permeabilization buffer. TfR intensity per cell was calculated as follows: (TfR signal × number of endosomes)/number of nuclei.

### Statistics.

All experiments were repeated three times to ensure reproducibility. Unless noted, data are shown as the means ± standard deviations (SDs) from three independent experiments. For microscopy, each experiment analyzed at least 50 YCVs, 50 individual cells, or 25 fields, and power analyses were performed posthoc to ensure that appropriate sample sizes were analyzed. *P* values were calculated using Student’s *t* test or one-way analysis of variance (ANOVA), with appropriate posthoc testing, using GraphPad Prism software.

## References

[1] Perry RD, Fetherston JD 1997 *Yersinia pestis*—etiologic agent of plague. Clin Microbiol Rev 10:35–66. 8993858 10.1128/cmr.10.1.35 PMC172914

[2] Butler T 2013 Plague gives surprises in the first decade of the 21st century in the United States and worldwide. Am J Trop Med Hyg 89:788–793. doi:10.4269/ajtmh.13-0191. 24043686 PMC3795114

[3] St John AL, Ang WXG, Huang M-N, Kunder CA, Chan EW, Gunn MD, Abraham SN 2014 S1P-dependent trafficking of intracellular *Yersinia pestis* through lymph nodes establishes buboes and systemic infection. Immunity 41:440–450. doi:10.1016/j.immuni.2014.07.013. 25238098 PMC4440548

[4] Gonzalez RJ, Lane MC, Wagner NJ, Weening EH, Miller VL 2015 Dissemination of a highly virulent pathogen: tracking the early events that define infection. PLoS Pathog 11:e1004587. doi:10.1371/journal.ppat.1004587. 25611317 PMC4303270

[5] Kwit N, Kugeler K, Petersen J, Plante L, Yaglom H, Kramer V, Schwartz B, House J, Colton L, Feldpausch A, Drenzek C, Baumbach J, DiMenna M, Fisher E, Debess E, Buttke D, Weinburke M, Percy C, Schriefer M, Gage K, Mead P 2015 Human plague - United States, 2015. MMWR Morb Mortal Wkly Rep 64:918–919. 26313475 10.15585/mmwr.mm6433a6

[6] Kugeler KJ, Staples JE, Hinckley AF, Gage KL, Mead PS 2015 Epidemiology of human plague in the United States, 1900–2012. Emerg Infect Dis 21:16–22. doi:10.3201/eid2101.140564. 25529546 PMC4285253

[7] Inglesby TV, Dennis DT, Henderson DA, Bartlett JG, Ascher MS, Eitzen E, Fine AD, Friedlander AM, Hauer J, Koerner JF, Layton M, McDade J, Osterholm MT, O’Toole T, Parker G, Perl TM, Russell PK, Schoch-Spana M, Tonat K 2000 Plague as a biological weapon: medical and public health management. Working Group on Civilian Biodefense. JAMA 283:2281–2290. doi:10.1001/jama.283.17.2281. 10807389

[8] Hinnebusch BJ 2005 The evolution of flea-borne transmission in *Yersinia pestis*. Curr Issues Mol Biol 7:197–212. 16053250

[9] Hinnebusch BJ, Perry RD, Schwan TG 1996 Role of the *Yersinia pestis* hemin storage (hms) locus in the transmission of plague by fleas. Science 273:367–370. doi:10.1126/science.273.5273.367. 8662526

[10] Paskewitz SM 1997 Transmission factors for insect-vectored microorganisms. Trends Microbiol 5:171–173. doi:10.1016/S0966-842X(97)01026-3. 9160500

[11] Hinnebusch BJ 2012 Biofilm-dependent and biofilm-independent mechanisms of transmission of *Yersinia pestis* by fleas. Adv Exp Med Biol 954:237–243. doi:10.1007/978-1-4614-3561-7_30. 22782769

[12] Chouikha I, Hinnebusch BJ 2012 *Yersinia*–flea interactions and the evolution of the arthropod-borne transmission route of plague. Curr Opin Microbiol 15:239–246. doi:10.1016/j.mib.2012.02.003. 22406208 PMC3386424

[13] Vadyvaloo V, Jarrett C, Sturdevant DE, Sebbane F, Hinnebusch BJ 2010 Transit through the flea vector induces a pretransmission innate immunity resistance phenotype in *Yersinia pestis*. PLoS Pathog 6:e1000783. doi:10.1371/journal.ppat.1000783. 20195507 PMC2829055

[14] Plano GV, Schesser K 2013 The *Yersinia pestis* type III secretion system: expression, assembly and role in the evasion of host defenses. Immunol Res 57:237–245. doi:10.1007/s12026-013-8454-3. 24198067

[15] Spinner JL, Winfree S, Starr T, Shannon JG, Nair V, Steele-Mortimer O, Hinnebusch BJ 2014 Yersinia pestis survival and replication within human neutrophil phagosomes and uptake of infected neutrophils by macrophages. J Leukoc Biol 95:389–398. doi:10.1189/jlb.1112551. 24227798 PMC3923079

[16] Straley SC, Harmon PA 1984 Growth in mouse peritoneal macrophages of *Yersinia pestis* lacking established virulence determinants. Infect Immun 45:649–654. 6469351 10.1128/iai.45.3.649-654.1984 PMC263344

[17] Shannon JG, Bosio CF, Hinnebusch BJ 2015 Dermal neutrophil, macrophage and dendritic cell responses to *Yersinia pestis* transmitted by fleas. PLoS Pathog 11:e1004734. doi:10.1371/journal.ppat.1004734. 25781984 PMC4363629

[18] Spinner JL, Hinnebusch BJ 2012 The life stage of *Yersinia pestis* in the flea vector confers increased resistance to phagocytosis and killing by murine polymorphonuclear leukocytes. Adv Exp Med Biol 954:159–163. doi:10.1007/978-1-4614-3561-7_20. 22782759

[19] Burrows TW, Bacon GA 1956 The basis of virulence in *Pasteurella pestis*: the development of resistance to phagocytosis in *vitro*. Br J Exp Pathol 37:286–299. 13342354 PMC2082155

[20] Vagima Y, Zauberman A, Levy Y, Gur D, Tidhar A, Aftalion M, Shafferman A, Mamroud E 2015 Circumventing *Y. pestis* virulence by early recruitment of neutrophils to the lungs during pneumonic plague. PLoS Pathog 11:e1004893. doi:10.1371/journal.ppat.1004893. 25974210 PMC4431741

[21] Lukaszewski RA, Kenny DJ, Taylor R, Rees DG, Hartley MG, Oyston PC 2005 Pathogenesis of *Yersinia pestis* infection in BALB/c mice: effects on host macrophages and neutrophils. Infect Immun 73:7142–7150. doi:10.1128/IAI.73.11.7142-7150.2005. 16239508 PMC1273833

[22] Janssen WA, Surgalla MJ 1969 Plague bacillus: survival within host phagocytes. Science 163:950–952. doi:10.1126/science.163.3870.950. 5763880

[23] Grabenstein JP, Fukuto HS, Palmer LE, Bliska JB 2006 Characterization of phagosome trafficking and identification of PhoP-regulated genes important for survival of *Yersinia pestis* in macrophages. Infect Immun 74:3727–3741. doi:10.1128/IAI.00255-06. 16790745 PMC1489716

[24] Grabenstein JP, Marceau M, Pujol C, Simonet M, Bliska JB 2004 The response regulator PhoP of *Yersinia pseudotuberculosis* is important for replication in macrophages and for virulence. Infect Immun 72:4973–4984. doi:10.1128/IAI.72.9.4973-4984.2004. 15321989 PMC517447

[25] Pujol C, Bliska JB 2003 The ability to replicate in macrophages is conserved between *Yersinia pestis* and *Yersinia pseudotuberculosis*. Infect Immun 71:5892–5899. doi:10.1128/IAI.71.10.5892-5899.2003. 14500510 PMC201058

[26] Pujol C, Grabenstein JP, Perry RD, Bliska JB 2005 Replication of *Yersinia pestis* in interferon gamma-activated macrophages requires ripA, a gene encoded in the pigmentation locus. Proc Natl Acad Sci U S A 102:12909–12914. doi:10.1073/pnas.0502849102. 16120681 PMC1200267

[27] Pujol C, Klein KA, Romanov GA, Palmer LE, Cirota C, Zhao Z, Bliska JB 2009 *Yersinia pestis* can reside in autophagosomes and avoid xenophagy in murine macrophages by preventing vacuole acidification. Infect Immun 77:2251–2261. doi:10.1128/IAI.00068-09. 19289509 PMC2687347

[28] Straley SC, Harmon PA 1984 *Yersinia pestis* grows within phagolysosomes in mouse peritoneal macrophages. Infect Immun 45:655–659. 6469352 10.1128/iai.45.3.655-659.1984 PMC263345

[29] Ye Z, Kerschen EJ, Cohen DA, Kaplan AM, van Rooijen N, Straley SC 2009 Gr1+ cells control growth of YopM-negative *Yersinia pestis* during systemic plague. Infect Immun 77:3791–3806. doi:10.1128/IAI.00284-09. 19581396 PMC2738001

[30] Bozue J, Mou S, Moody KL, Cote CK, Trevino S, Fritz D, Worsham P 2011 The role of the phoPQ operon in the pathogenesis of the fully virulent CO92 strain of *Yersinia pestis* and the IP32953 strain of *Yersinia pseudotuberculosis*. Microb Pathog 50:314–321. doi:10.1016/j.micpath.2011.02.005. 21320584

[31] Oyston PC, Dorrell N, Williams K, Li SR, Green M, Titball RW, Wren BW 2000 The response regulator PhoP is important for survival under conditions of macrophage-induced stress and virulence in *Yersinia pestis*. Infect Immun 68:3419–3425. doi:10.1128/IAI.68.6.3419-3425.2000. 10816493 PMC97616

[32] Rust JH Jr, Cavanaugh DC, O’Shita R, Marshall JD 1971 The role of domestic animals in the epidemiology of plague. I. Experimental infection of dogs and cats. J Infect Dis 124:522–526. doi:10.1093/infdis/124.5.522. 5115673

[33] Ponnusamy D, Clinkenbeard KD 2012 *Yersinia pestis* intracellular parasitism of macrophages from hosts exhibiting high and low severity of plague. PLoS One 7:e42211. doi:10.1371/journal.pone.0042211. 22848745 PMC3407133

[34] Pachulec E, Abdelwahed Bagga RB, Chevallier L, O’Donnell H, Guillas C, Jaubert J, Montagutelli X, Carniel E, Demeure CE 2017 Enhanced macrophage M1 polarization and resistance to apoptosis enable resistance to plague. J Infect Dis 216:761–770. doi:10.1093/infdis/jix348. 28934426

[35] Connor MG, Pulsifer AR, Price CT, Abu Kwaik Y, Lawrenz MB 2015 *Yersinia pestis* requires host Rab1b for survival in macrophages. PLoS Pathog 11:e1005241. doi:10.1371/journal.ppat.1005241. 26495854 PMC4619670

[36] Martinez O, Goud B 1998 Rab proteins. Biochim Biophys Acta 1404:101–112. doi:10.1016/S0167-4889(98)00050-0. 9714762

[37] Satoh A, Wang Y, Malsam J, Beard MB, Warren G 2003 Golgin-84 is a rab1 binding partner involved in Golgi structure. Traffic 4:153–161. doi:10.1034/j.1600-0854.2003.00103.x. 12656988 PMC3282115

[38] Kühbacher A, Emmenlauer M, Rämo P, Kafai N, Dehio C, Cossart P, Pizarro-Cerdá J 2015 Genome-wide siRNA screen identifies complementary signaling pathways involved in Listeria infection and reveals different actin nucleation mechanisms during Listeria cell invasion and actin comet tail formation. mBio 6:e00598-15. doi:10.1128/mBio.00598-15 25991686 PMC4442140

[39] McDonough JA, Newton HJ, Klum S, Swiss R, Agaisse H, Roy CR 2013 Host pathways important for *Coxiella burnetii* infection revealed by genome-wide RNA interference screening. mBio 4:e00606-12. doi:10.1128/mBio.00606-12. 23362322 PMC3560531

[40] Ooi YS, Stiles KM, Liu CY, Taylor GM, Kielian M 2013 Genome-wide RNAi screen identifies novel host proteins required for alphavirus entry. PLoS Pathog 9:e1003835. doi:10.1371/journal.ppat.1003835. 24367265 PMC3868536

[41] Thornbrough JM, Hundley T, Valdivia R, Worley MJ 2012 Human genome-wide RNAi screen for host factors that modulate intracellular Salmonella growth. PLoS One 7:e38097. doi:10.1371/journal.pone.0038097. 22701604 PMC3372477

[42] Akimana C, Al-Khodor S, Abu Kwaik Y 2010 Host factors required for modulation of phagosome biogenesis and proliferation of *Francisella tularensis* within the cytosol. PLoS One 5:e11025. doi:10.1371/journal.pone.0011025. 20552012 PMC2883998

[43] Kumar D, Nath L, Kamal MA, Varshney A, Jain A, Singh S, Rao KV 2010 Genome-wide analysis of the host intracellular network that regulates survival of *Mycobacterium tuberculosis*. Cell 140:731–743. doi:10.1016/j.cell.2010.02.012. 20211141

[44] Jayaswal S, Kamal MA, Dua R, Gupta S, Majumdar T, Das G, Kumar D, Rao KV 2010 Identification of host-dependent survival factors for intracellular Mycobacterium tuberculosis through an siRNA screen. PLoS Pathog 6:e1000839. doi:10.1371/journal.ppat.1000839. 20419122 PMC2855445

[45] Qin QM, Pei J, Ancona V, Shaw BD, Ficht TA, de Figueiredo P 2008 RNAi screen of endoplasmic reticulum-associated host factors reveals a role for IRE1alpha in supporting *Brucella* replication. PLoS Pathog 4:e1000110. doi:10.1371/journal.ppat.1000110. 18654626 PMC2453327

[46] Krishnan MN, Ng A, Sukumaran B, Gilfoy FD, Uchil PD, Sultana H, Brass AL, Adametz R, Tsui M, Qian F, Montgomery RR, Lev S, Mason PW, Koski RA, Elledge SJ, Xavier RJ, Agaisse H, Fikrig E 2008 RNA interference screen for human genes associated with West Nile virus infection. Nature 455:242–245. doi:10.1038/nature07207. 18690214 PMC3136529

[47] Derré I, Pypaert M, Dautry-Varsat A, Agaisse H 2007 RNAi screen in Drosophila cells reveals the involvement of the Tom complex in Chlamydia infection. PLoS Pathog 3:1446–1458. doi:10.1371/journal.ppat.0030155. 17967059 PMC2042019

[48] Agaisse H, Burrack LS, Philips JA, Rubin EJ, Perrimon N, Higgins DE 2005 Genome-wide RNAi screen for host factors required for intracellular bacterial infection. Science 309:1248–1251. doi:10.1126/science.1116008. 16020693

[49] Philips JA, Rubin EJ, Perrimon N 2005 Drosophila RNAi screen reveals CD36 family member required for mycobacterial infection. Science 309:1251–1253. doi:10.1126/science.1116006. 16020694

[50] Sun Y, Connor MG, Pennington JM, Lawrenz MB 2012 Development of bioluminescent bioreporters for *in vitro* and *in vivo* tracking of *Yersinia pestis*. PLoS One 7:e47123. doi:10.1371/journal.pone.0047123. 23071730 PMC3469486

[51] Stein MP, Müller MP, Wandinger-Ness A 2012 Bacterial pathogens commandeer Rab GTPases to establish intracellular niches. Traffic 13:1565–1588. doi:10.1111/tra.12000. 22901006 PMC3530015

[52] Bhuin T, Roy JK 2014 Rab proteins: the key regulators of intracellular vesicle transport. Exp Cell Res 328:1–19. doi:10.1016/j.yexcr.2014.07.027. 25088255

[53] Grant BD, Donaldson JG 2009 Pathways and mechanisms of endocytic recycling. Nat Rev Mol Cell Biol 10:597–608. doi:10.1038/nrm2755. 19696797 PMC3038567

[54] Li F, Vierstra RD 2012 Autophagy: a multifaceted intracellular system for bulk and selective recycling. Trends Plant Sci 17:526–537. doi:10.1016/j.tplants.2012.05.006. 22694835

[55] Luzio JP, Piper SC, Bowers K, Parkinson MD, Lehner PJ, Bright NA 2009 ESCRT proteins and the regulation of endocytic delivery to lysosomes. Biochem Soc Trans 37:178–180. doi:10.1042/BST0370178. 19143626

[56] Luzio JP, Pryor PR, Gray SR, Gratian MJ, Piper RC, Bright NA 2005 Membrane traffic to and from lysosomes. Biochem Soc Symp 72:77–86. doi:10.1042/bss0720077. 15649132

[57] Kinchen JM, Ravichandran KS 2008 Phagosome maturation: going through the acid test. Nat Rev Mol Cell Biol 9:781–795. doi:10.1038/nrm2515. 18813294 PMC2908392

[58] Seto S, Tsujimura K, Koide Y 2011 Rab GTPases regulating phagosome maturation are differentially recruited to mycobacterial phagosomes. Traffic 12:407–420. doi:10.1111/j.1600-0854.2011.01165.x. 21255211

[59] Huang B, Hubber A, McDonough JA, Roy CR, Scidmore MA, Carlyon JA 2010 The *Anaplasma phagocytophilum*-occupied vacuole selectively recruits Rab-GTPases that are predominantly associated with recycling endosomes. Cell Microbiol 12:1292–1307. doi:10.1111/j.1462-5822.2010.01468.x. 20345488 PMC2923681

[60] Rzomp KA, Scholtes LD, Briggs BJ, Whittaker GR, Scidmore MA 2003 Rab GTPases are recruited to chlamydial inclusions in both a species-dependent and species-independent manner. Infect Immun 71:5855–5870. doi:10.1128/IAI.71.10.5855-5870.2003. 14500507 PMC201052

[61] Rejman Lipinski A, Heymann J, Meissner C, Karlas A, Brinkmann V, Meyer TF, Heuer D 2009 Rab6 and Rab11 regulate *Chlamydia trachomatis* development and golgin-84-dependent Golgi fragmentation. PLoS Pathog 5:e1000615. doi:10.1371/journal.ppat.1000615. 19816566 PMC2752117

[62] Longatti A, Lamb CA, Razi M, Yoshimura S, Barr FA, Tooze SA 2012 TBC1D14 regulates autophagosome formation via Rab11- and ULK1-positive recycling endosomes. J Cell Biol 197:659–675. doi:10.1083/jcb.201111079. 22613832 PMC3365497

[63] Longatti A, Tooze SA 2012 Recycling endosomes contribute to autophagosome formation. Autophagy 8:1682–1683. doi:10.4161/auto.21486. 22874560 PMC3494599

[64] Szatmári Z, Kis V, Lippai M, Hegedus K, Faragó T, Lorincz P, Tanaka T, Juhász G, Sass M 2014 Rab11 facilitates cross-talk between autophagy and endosomal pathway through regulation of Hook localization. Mol Biol Cell 25:522–531. doi:10.1091/mbc.E13-10-0574. 24356450 PMC3923643

[65] Ligeon LA, Moreau K, Barois N, Bongiovanni A, Lacorre DA, Werkmeister E, Proux-Gillardeaux V, Galli T, Lafont F 2014 Role of VAMP3 and VAMP7 in the commitment of *Yersinia pseudotuberculosis* to LC3-associated pathways involving single- or double-membrane vacuoles. Autophagy 10:1588–1602. doi:10.4161/auto.29411. 25046114 PMC4206537

[66] Moreau K, Lacas-Gervais S, Fujita N, Sebbane F, Yoshimori T, Simonet M, Lafont F 2010 Autophagosomes can support *Yersinia pseudotuberculosis* replication in macrophages. Cell Microbiol 12:1108–1123. doi:10.1111/j.1462-5822.2010.01456.x. 20180800

[67] Ray A, Dittel BN 2010 Isolation of mouse peritoneal cavity cells. J Vis Exp 35 doi:10.3791/1488. PMC3152216 20110936

[68] Welsh CT, Summersgill JT, Miller RD 2004 Increases in c-Jun N-terminal kinase/stress-activated protein kinase and p38 activity in monocyte-derived macrophages following the uptake of *Legionella pneumophila*. Infect Immun 72:1512–1518. doi:10.1128/IAI.72.3.1512-1518.2004. 14977957 PMC356002

[69] Olakanmi O, Britigan BE, Schlesinger LS 2000 Gallium disrupts iron metabolism of mycobacteria residing within human macrophages. Infect Immun 68:5619–5627. doi:10.1128/IAI.68.10.5619-5627.2000. 10992462 PMC101514

[70] Santic M, Molmeret M, Abu Kwaik Y 2005 Maturation of the *Legionella pneumophila*-containing phagosome into a phagolysosome within gamma interferon-activated macrophages. Infect Immun 73:3166–3171. doi:10.1128/IAI.73.5.3166-3171.2005. 15845527 PMC1087382

[71] Doll JM, Zeitz PS, Ettestad P, Bucholtz AL, Davis T, Gage K 1994 Cat-transmitted fatal pneumonic plague in a person who traveled from Colorado to Arizona. Am J Trop Med Hyg 51:109–114. doi:10.4269/ajtmh.1994.51.109. 8059908

[72] Galen JE, Nair J, Wang JY, Wasserman SS, Tanner MK, Sztein MB, Levine MM 1999 Optimization of plasmid maintenance in the attenuated live vector vaccine strain *Salmonella typhi* CVD 908-htrA. Infect Immun 67:6424–6433. 10569759 10.1128/iai.67.12.6424-6433.1999 PMC97051

[73] Livak KJ, Schmittgen TD 2001 Analysis of relative gene expression data using real-time quantitative PCR and the 2(−Delta Delta C(T)) method. Methods 25:402–408. doi:10.1006/meth.2001.1262. 11846609

[74] Seto S, Matsumoto S, Ohta I, Tsujimura K, Koide Y 2009 Dissection of Rab7 localization on *Mycobacterium tuberculosis* phagosome. Biochem Biophys Res Commun 387:272–277. doi:10.1016/j.bbrc.2009.06.152. 19580780

[75] Stark C, Breitkreutz BJ, Reguly T, Boucher L, Breitkreutz A, Tyers M 2006 BioGRID: a general repository for interaction datasets. Nucleic Acids Res 34:D535–D539. doi:10.1093/nar/gkj109. 16381927 PMC1347471

[76] Szklarczyk D, Franceschini A, Wyder S, Forslund K, Heller D, Huerta-Cepas J, Simonovic M, Roth A, Santos A, Tsafou KP, Kuhn M, Bork P, Jensen LJ, von Mering C 2015 STRING v10: protein-protein interaction networks, integrated over the tree of life. Nucleic Acids Res 43:D447–D452. doi:10.1093/nar/gku1003. 25352553 PMC4383874

[77] Shannon P, Markiel A, Ozier O, Baliga NS, Wang JT, Ramage D, Amin N, Schwikowski B, Ideker T 2003 Cytoscape: a software environment for integrated models of biomolecular interaction networks. Genome Res 13:2498–2504. doi:10.1101/gr.1239303. 14597658 PMC403769

[78] Bindea G, Mlecnik B, Hackl H, Charoentong P, Tosolini M, Kirilovsky A, Fridman WH, Pagès F, Trajanoski Z, Galon J 2009 ClueGO: a Cytoscape plug-in to decipher functionally grouped gene ontology and pathway annotation networks. Bioinformatics 25:1091–1093. doi:10.1093/bioinformatics/btp101. 19237447 PMC2666812

[79] Bindea G, Galon J, Mlecnik B 2013 CluePedia Cytoscape plugin: pathway insights using integrated experimental and *in silico* data. Bioinformatics 29:661–663. doi:10.1093/bioinformatics/btt019. 23325622 PMC3582273

[80] Schindelin J, Arganda-Carreras I, Frise E, Kaynig V, Longair M, Pietzsch T, Preibisch S, Rueden C, Saalfeld S, Schmid B, Tinevez JY, White DJ, Hartenstein V, Eliceiri K, Tomancak P, Cardona A 2012 Fiji: an open-source platform for biological-image analysis. Nat Methods 9:676–682. doi:10.1038/nmeth.2019. 22743772 PMC3855844

