# Supplementary material for: Yersinia pestis Targets the Host Endosome Recycling Pathway during the Biogenesis of the Yersinia-Containing Vacuole To Avoid Killing by Macrophages
Source: mBio. 2018 Feb 20;9(1):e01800-17. doi: 10.1128/mBio.01800-17 (PMC5821078; doi:10.1128/mBio.01800-17)
Supplement: TABLE S1 [file mbo001183734st1.pdf]

Table S1. Validated hits from whole genome screen.			
**Entrez Gene ID**	**Gene Symbol**	**MGI Gene/Marker ID**	**Ensembl ID**
66409	Rsl1d1	MGI:1913659	ENSMUSG00000005846
74116	Pi16	MGI:1921366	ENSMUSG00000024011
20336	Exoc4	MGI:1096376	ENSMUSG00000029763
21787	Tfg	MGI:1338041	ENSMUSG00000022757
54633	Pqbp1	MGI:1859638	ENSMUSG00000031157
83672	Sytl3	MGI:1933367	ENSMUSG00000041831
209456	Trp53bp2	MGI:2138319	ENSMUSG00000026510
218952	Fermt2	MGI:2385001	ENSMUSG00000037712
14064	F2rl2	MGI:1298208	ENSMUSG00000021675
56284	Mrpl19	MGI:1926274	ENSMUSG00000030045
213556	Plekhh2	MGI:2146813	ENSMUSG00000040852
235330	Ttc12	MGI:2444588	ENSMUSG00000040219
74143	Opa1	MGI:1921393	ENSMUSG00000038084
98685	Trmt1l	MGI:1916185	ENSMUSG00000053286
56096	Plac1	MGI:1926287	ENSMUSG00000061082
74782	Glt8d2	MGI:1922032	ENSMUSG00000020251
56523	Pmfbp1	MGI:1930136	ENSMUSG00000031727
56522	Papolb	MGI:1932115	ENSMUSG00000074817
233489	Picalm	MGI:2385902	ENSMUSG00000039361
22110	Tspyl1	MGI:1298395	ENSMUSG00000047514
208177	Phldb2	MGI:2444981	ENSMUSG00000033149
16174	Il18rap	MGI:1338888	ENSMUSG00000026068
19340	Rab3d	MGI:97844	ENSMUSG00000019066
17527	Mpv17	MGI:97138	ENSMUSG00000090262
18143	Npas2	MGI:109232	ENSMUSG00000026077
76308	Rab1b	MGI:1923558	ENSMUSG00000024870
16678	Krt1	MGI:96698	ENSMUSG00000046834
66681	Pgm1	MGI:97564	ENSMUSG00000029171
215335	Slc36a1	MGI:2445299	ENSMUSG00000020261
14997	H2-M9	MGI:1276570	ENSMUSG00000067201
18641	Pfkl	MGI:97547	ENSMUSG00000020277
19331	Rab19	MGI:103292	ENSMUSG00000029923
17289	Mertk	MGI:96965	ENSMUSG00000014361
71472	Usp19	MGI:1918722	ENSMUSG00000006676
75530	Lyrm7	MGI:1922780	ENSMUSG00000020268
18538	Pcna	MGI:97503	ENSMUSG00000027342
20333	Sec22b	MGI:1338759	ENSMUSG00000027879
227157	Mpp4	MGI:2386681	ENSMUSG00000079550
19332	Rab20	MGI:102789	ENSMUSG00000031504
231507	Plac8	MGI:2445289	ENSMUSG00000029322
18071	Nhlh1	MGI:98481	ENSMUSG00000051251
81500	Sil1	MGI:1932040	ENSMUSG00000024357
58234	Shank3	MGI:1930016	ENSMUSG00000022623
19341	Rab4a	MGI:105069	ENSMUSG00000019478
20438	Siah1b	MGI:108063	ENSMUSG00000040749
12266	C3	MGI:88227	ENSMUSG00000024164
327814	Ppfia2	MGI:2443834	ENSMUSG00000053825
67474	Snap29	MGI:1914724	ENSMUSG00000022765
170719	Oxr1	MGI:2179326	ENSMUSG00000022307
15387	Hnrnpk	MGI:99894	ENSMUSG00000021546
77604	Rbm12b2	MGI:1924854	ENSMUSG00000052137
238831	Ppwd1	MGI:2443069	ENSMUSG00000021713
76338	Rab2b	MGI:1923588	ENSMUSG00000022159
234852	Chmp1a	MGI:1920159	ENSMUSG00000000743
269198	Nbeal1	MGI:2444343	ENSMUSG00000073664
228775	Trib3	MGI:1345675	ENSMUSG00000032715
18690	Phxr5	MGI:104521	
74053	Grip1	MGI:1921303	ENSMUSG00000034813
268294	Zbtb24	MGI:3039618	ENSMUSG00000019826
15002	H2-Ob	MGI:95925	ENSMUSG00000041538
20195	S100a11	MGI:1338798	ENSMUSG00000027907
76366	Mtif3	MGI:1923616	ENSMUSG00000016510
18530	Pcdh8	MGI:1306800	ENSMUSG00000036422
259100	Olfr666	MGI:3030500	ENSMUSG00000063582
19110	Prl4a1	MGI:1206587	ENSMUSG00000005891
18720	Pip5k1a	MGI:107929	ENSMUSG00000028126
104776	Aldh6a1	MGI:1915077	ENSMUSG00000021238
241035	Pkhd1	MGI:2155808	ENSMUSG00000043760
19053	Ppp2cb	MGI:1321161	ENSMUSG00000009630
18181	Nrf1	MGI:1332235	ENSMUSG00000058440
18744	Pja1	MGI:1101765	ENSMUSG00000034403
