# Supplementary figures and images for: Yersinia pestis Targets the Host Endosome Recycling Pathway during the Biogenesis of the Yersinia-Containing Vacuole To Avoid Killing by Macrophages
Source: mBio. 2018 Feb 20;9(1):e01800-17. doi: 10.1128/mBio.01800-17 (PMC5821078; doi:10.1128/mBio.01800-17)

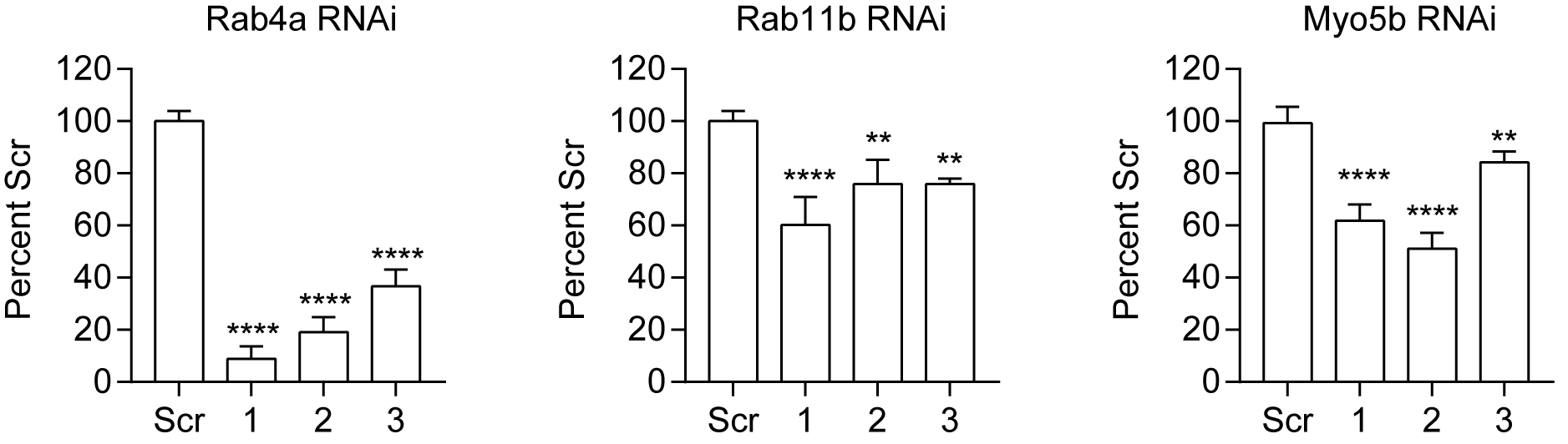

Supplement: FIG S1 [file mbo001183734sf1.tif]

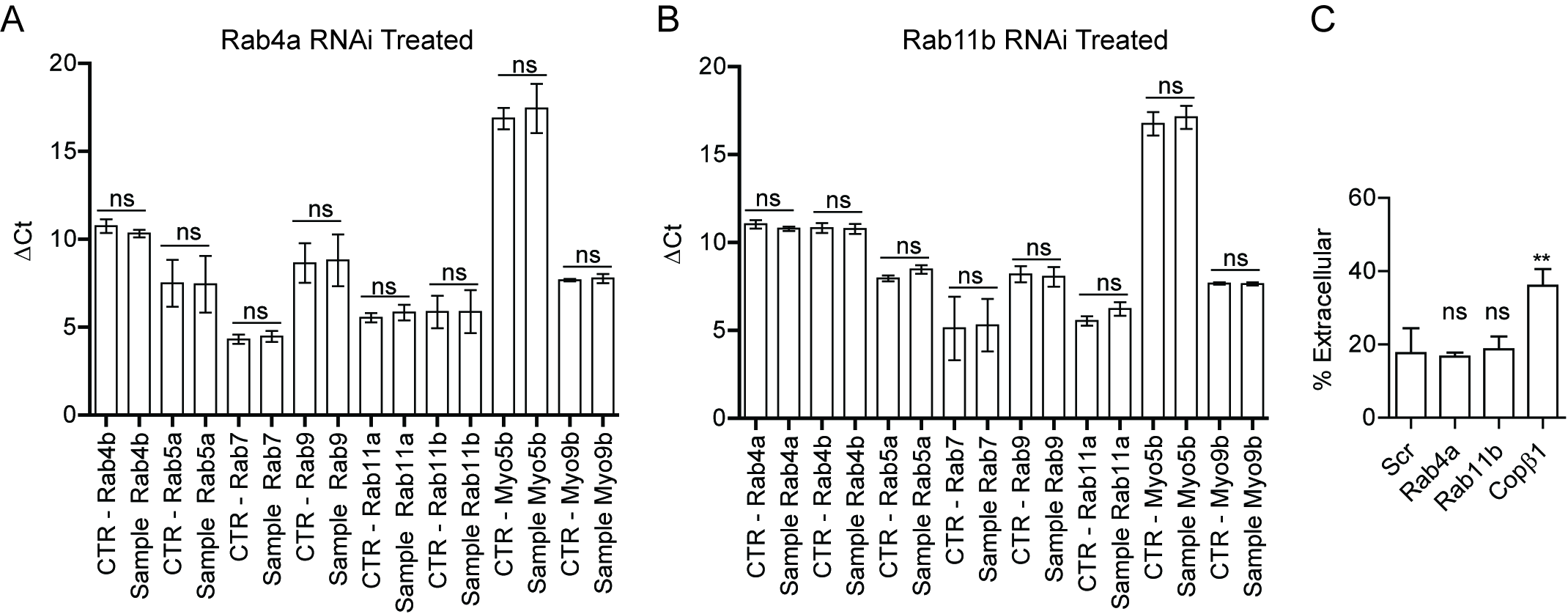

Supplement: FIG S2 [file mbo001183734sf2.tif]

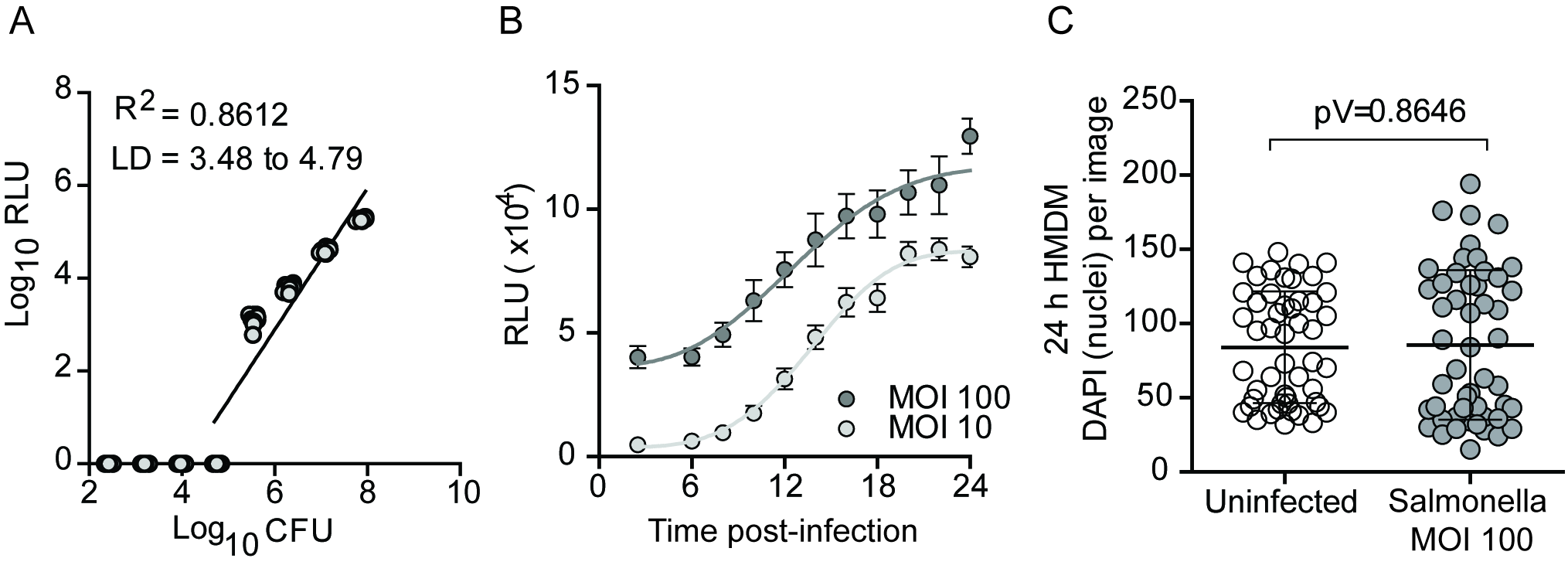

Supplement: FIG S3 [file mbo001183734sf3.tif]

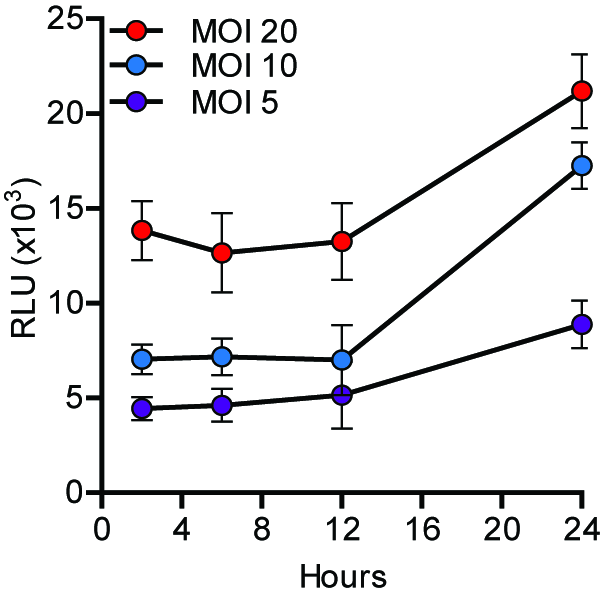

Supplement: FIG S4 [file mbo001183734sf4.tif]

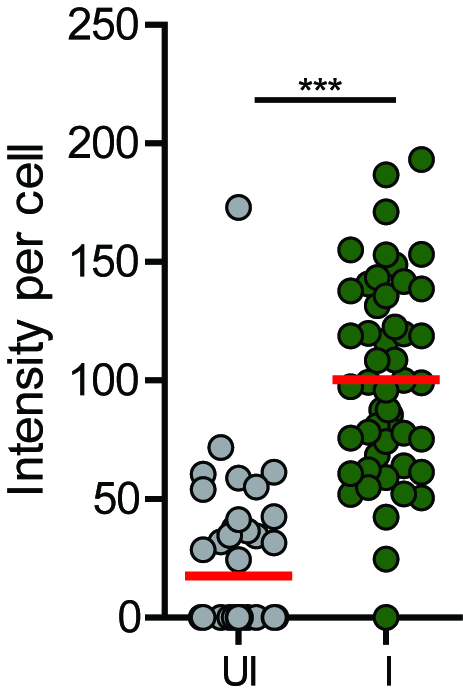

Supplement: FIG S5 [file mbo001183734sf5.tif]

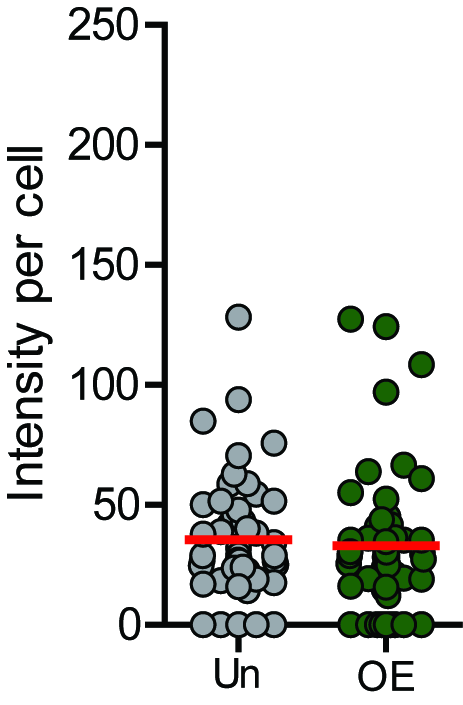

Supplement: FIG S6 [file mbo001183734sf6.tif]
